# Impact of COVID-19 vaccination coverage on global disability burden of Guillain-Barré syndrome

**DOI:** 10.1038/s41541-025-01239-1

**Published:** 2025-08-02

**Authors:** Xīn Gào, Chen Zhao, Junting Yang, Ziming Yang, Jingnan Feng, Siyan Zhan, Dongsheng Fan, Zhike Liu

**Affiliations:** 1 Department of Epidemiology and Biostatistics, School of Public Health, Peking University, Beijing, China; 2 Key Laboratory of Epidemiology of Major Diseases, Peking University, Ministry of Education, Beijing, China; 3 Department of Neurology, Peking University Third Hospital, Beijing, China; 4 Beijing Key Laboratory of Biomarker and Translational Research in Neurodegenerative Diseases, Beijing, China; 5 Key Laboratory for Neuroscience, National Health Commission/Ministry of Education, Peking University, Beijing, China; 6 Peking University First Hospital, Beijing, China; 7 Center for Intelligent Public Health, Institute for Artificial Intelligence, Peking University, Beijing, China; 8 Research Center of Clinical Epidemiology, Peking University Third Hospital, Beijing, China

**Keywords:** Vaccines, Neurological disorders, Epidemiology

## Abstract

The global burden of Guillain-Barré syndrome (GBS), an immune-mediated neuropathy, remains poorly characterized during the COVID-19 pandemic. We analyzed age-standardized years lived with disability (YLD) for GBS from 1990 to 2021 using GBD 2021 data and COVID-19 vaccination coverage from Our World in Data, focusing on 2020–2021. During the pandemic, GBS YLD rates rose dramatically, with greater increases seen in low-SDI regions, females and individuals aged 15–29 years. Higher vaccination coverage was inversely associated with GBS disability burden, exhibiting a non-linear protective effect at moderate to high coverage levels. Causal mediation analysis indicated that 44.6% of this association was mediated by reductions in COVID-19 incidence, highlighting both direct and indirect neuroprotective benefits of vaccination programs. These results underscore the importance of sustaining and expanding the vaccine rollout to mitigate the secondary neurological burden associated with emerging infections.

## Introduction

Guillain-Barré syndrome (GBS) is an acute-onset autoimmune polyradiculoneuropathy, which is the most common cause of acute flaccid paralysis worldwide^1^. This condition can lead to life-threatening motor weakness and up to 30% of patients require admission to the intensive care unit and mechanical ventilation due to respiratory failure^2^. Approximately two-thirds of GBS cases present antecedent infections, mostly respiratory and gastrointestinal infections^3^. The prevalence of GBS can show a transient rise in outbreaks of certain infectious diseases. An example is the surge of GBS following the 2015–2016 Zika virus outbreak in Latin America and the Caribbean, with a 2.6-fold increase in incidence above the background rate^4^. More recently, the severe acute respiratory syndrome coronavirus 2 (SARS-CoV-2) caused a global pandemic, and some studies suggest a potential link between SARS-CoV-2 infection and GBS^5,6^. However, existing studies are largely limited to smaller populations, and comprehensive estimates of the global GBS burden during the pandemic remain lacking.

As one of the most cost-effective and efficient measures for COVID-19 prevention and control, vaccination has raised public concerns about the safety, particularly regarding the risk of triggering GBS^7^. Previous studies have shown a higher incidence of GBS following adenovirus-based vaccines, while mRNA-based vaccines have not demonstrated this association^8–11^. On the other hand, vaccination has the potential to reduce the burden of GBS by preventing SARS-CoV-2 transmission and infection. Therefore, it is urged to investigate the benefits and risks at the population level to provide evidence-based support for immunization policy decisions. To the best of our knowledge, the overall impact of global vaccination efforts on the GBS burden has not been investigated.

Although the Global Burden of Disease, Injuries, and Risk Factors Study (GBD) 2021 has recently updated the prevalence and years lived with disability (YLDs) of GBS, the results did not account for GBS cases attributable to COVID-19. Instead, the study grouped GBS and persistent cognitive symptoms following COVID-19 under the category of neurological complications related to COVID-19 for pooled analysis^12^. This study aimed to conduct a comprehensive analysis of the global, regional, and national prevalence and YLDs of GBS across all age groups, sexes, and causes, between 1990 and 2021 with a particular focus on the first two years of the COVID-19 pandemic. Furthermore, we explored public health measures, including vaccination and non-pharmacological interventions, that might influence the GBS burden during the pandemic.

## Results

### Spatial distribution and temporal trends

Globally, 230.1 thousand (95% UI: 194.3 to 269.7) and 471.9 thousand (95% UI: 389.2 to 554.1) individuals were affected by GBS in 2020 and 2021, respectively (Supplementary Table 1). GBS accounted for 68.1 thousand (95% UI: 44.8 to 98.6) YLDs in 2020, with an age-standardized YLD rate of 0.86 (95% UI: 0.56 to 1.24) per 100,000 people, and 139.6 thousand (95% UI: 90.4 to 202.4) YLDs in 2021, with an age-standardized YLD rate of 1.75 (95% UI: 1.12 to 2.54) per 100,000 people (Table 1). The EAPCs in age-standardized YLD rate were 70.35% (95% UI: 38.73‒109.06) for the period from 2019 to 2021, and were 0.16% (95% UI: 0.14 to 0.18) over the longer period from 1990 to 2019 (Table 1).

**Table 1:** Years lived with disability of Guillain-Barré syndrome in 2020 and 2021 and the estimated annual percentage change in the age-standardized rates, at global, regional and national levels, from 1990 to 2021

GBD super regions	GBD regions	2020		2021		2019–2021	1990–2019
		Count (thousands)	ASYR (per 100,000 people)	Count (thousands)	ASYR (per 100,000 people)	EAPC (%) in ASYR	EAPC (%) in ASYR
Global		68.1 (44.8 to 98.6)	0.86 (0.56 to 1.24)	139.6 (90.4 to 202.4)	1.75 (1.12 to 2.54)	70.30 (38.73 to 109.06)	0.16 (0.14 to 0.18)
Low SDI		9.9 (6.3 to 14.5)	1.00 (0.64 to 1.46)	24.4 (15.1 to 35.2)	2.37 (1.49 to 3.44)	103.21 (71.10 to 141.35)	−0.01 (−0.02 to 0)
Low-middle SDI		19.1 (12.4 to 27.7)	1.05 (0.69 to 1.52)	43.6 (27.5 to 64.3)	2.32 (1.46 to 3.42)	88.23 (56.64 to 126.19)	0.02 (0.01 to 0.03)
Middle SDI		18 (11.9 to 26)	0.73 (0.48 to 1.06)	36.8 (23.6 to 53.5)	1.48 (0.95 to 2.15)	68.62 (36.06 to 108.96)	0.42 (0.4 to 0.44)
High-middle SDI		7.6 (4.9 to 10.8)	0.53 (0.35 to 0.77)	15.8 (10.2 to 22.8)	1.13 (0.72 to 1.63)	66.93 (26.53 to 120.23)	0.11 (0.09 to 0.14)
High SDI		13.6 (8.9 to 19.3)	1.02 (0.67 to 1.46)	18.9 (12.5 to 26.4)	1.48 (0.98 to 2.10)	28.02 (11.46 to 47.03)	0.11 (0.06 to 0.16)
Central Europe, eastern Europe, and central Asia		3.6 (2.3 to 5.1)	0.79 (0.51 to 1.14)	10.3 (6.6 to 15.2)	2.29 (1.46 to 3.35)	113.17 (49.73 to 203.50)	−0.01 (−0.02 to 0)
	Central Asia	0.8 (0.5 to 1.2)	0.90 (0.58 to 1.30)	2.0 (1.2 to 2.9)	2.06 (1.27 to 3.01)	94.77 (61.82 to 134.42)	0.01 (0 to 0.01)
	Central Europe	0.8 (0.5 to 1.1)	0.57 (0.37 to 0.83)	2.7 (1.7 to 4.0)	2.14 (1.33 to 3.12)	129.00 (30.68 to 301.28)	−0.14 (−0.18 to −0.1)
	Eastern Europe	2 (1.3 to 2.9)	0.86 (0.55 to 1.25)	5.6 (3.6 to 8.5)	2.49 (1.58 to 3.75)	113.75 (51.05 to 202.47)	0.01 (0.01 to 0.01)
Hign-income		14.8 (9.7 to 21)	1.11 (0.73 to 1.59)	20.1 (13.3 to 28.2)	1.57 (1.04 to 2.23)	25.99 (10.67 to 43.43)	0.28 (0.22 to 0.33)
	Australasia	0.2 (0.1 to 0.3)	0.47 (0.30 to 0.69)	0.2 (0.1 to 0.3)	0.48 (0.31 to 0.7)	1.52 (1.01 to 2.04)	1.24 (1.02 to 1.46)
	High-income Asia Pacific	3.3 (2.1 to 4.8)	1.79 (1.15 to 2.63)	3.4 (2.3 to 5.0)	1.88 (1.22 to 2.77)	2.84 (0.31 to 5.43)	0.15 (0.10 to 0.20)
	High-income North America	6.7 (4.4 to 9.5)	1.38 (0.91 to 1.95)	9.3 (6.1 to 13)	2.05 (1.34 to 2.92)	30.44 (12.73 to 50.94)	0.45 (0.33 to 0.56)
	Southern Latin America	0.9 (0.6 to 1.3)	1.25 (0.82 to 1.83)	1.3 (0.8 to 1.8)	1.79 (1.14 to 2.62)	25.65 (8.45 to 45.59)	0.24 (0.16 to 0.32)
	Western Europe	3.8 (2.5 to 5.5)	0.69 (0.46 to 0.98)	5.9 (3.9 to 8.3)	1.13 (0.74 to 1.61)	42.26 (21.45 to 66.64)	0.71 (0.66 to 0.76)
Latin America and Caribbean		7.3 (4.8 to 10.4)	1.23 (0.81 to 1.76)	14.1 (9 to 20.5)	2.35 (1.49 to 3.41)	67.94 (45.27 to 94.15)	0.11 (0.06 to 0.16)
	Andean Latin America	0.9 (0.6 to 1.3)	1.41 (0.92 to 2.06)	1.7 (1.1 to 2.6)	2.64 (1.65 to 3.90)	86.68 (85.54 to 87.83)	−0.17 (−0.21 to −0.13)
	Caribbean	0.4 (0.2 to 0.5)	0.76 (0.49 to 1.11)	0.6 (0.4 to 0.8)	1.15 (0.73 to 1.65)	36.59 (22.28 to 52.57)	0.02 (−0.1 to 0.15)
	Tropical Latin America	1.9 (1.2 to 2.7)	0.80 (0.52 to 1.14)	4.7 (3.0 to 6.9)	2.00 (1.27 to 2.91)	124.04 (98.19 to 153.26)	0.04 (−0.04 to 0.12)
	Central Latin America	4.1 (2.7 to 5.9)	1.66 (1.09 to 2.37)	7.1 (4.5 to 10.2)	2.81 (1.78 to 4.05)	48.24 (27.37 to 72.54)	0.06 (0.02 to 0.10)
North Africa and Middle East	North Africa and Middle East	5.4 (3.4 to 7.8)	0.91 (0.58 to 1.33)	11.5 (7.2 to 16.8)	1.91 (1.19 to 2.79)	98.62 (87.34 to 110.58)	0.04 (0.02 to 0.06)
South Asia	South Asia	19.6 (12.9 to 28.9)	1.10 (0.73 to 1.62)	45.9 (28.8 to 68.5)	2.51 (1.58 to 3.75)	85.89 (47.92 to 133.6)	0 (0 to 0)
Southeast Asia, east Asia, and Oceania		7.6 (4.8 to 11.4)	0.34 (0.22 to 0.52)	12.8 (8.2 to 18.5)	0.60 (0.38 to 0.86)	39.58 (7.99 to 80.41)	0.45 (0.37 to 0.52)
	Southeast Asia	4.6 (2.9 to 6.7)	0.66 (0.42 to 0.97)	9.7 (6.1 to 14.0)	1.39 (0.88 to 2.00)	55.86 (10.41 to 120.03)	0.09 (0.07 to 0.1)
	East Asia	3 (1.8 to 4.5)	0.19 (0.12 to 0.30)	2.9 (1.8 to 4.5)	0.19 (0.12 to 0.29)	0.39 (−2.77 to 3.66)	0.32 (0.13 to 0.5)
	Oceania	0 (0 to 0.1)	0.39 (0.25 to 0.60)	0.1 (0.1 to 0.2)	0.95 (0.55 to 1.54)	59.26 (−1.75 to 158.14)	0 (0 to 0)
Sub-Saharan Africa		9.9 (6.3 to 14.5)	0.99 (0.64 to 1.44)	24.9 (15.7 to 36.5)	2.40 (1.52 to 3.49)	112.58 (84 to 145.6)	0.02 (0.01 to 0.02)
	Western Sub-Saharan Africa	5.0 (3.2 to 7.3)	1.17 (0.76 to 1.69)	10.1 (6.4 to 15.0)	2.31 (1.48 to 3.40)	100.84 (95.87 to 105.93)	−0.01 (−0.02 to −0.01)
	Eastern Sub-Saharan Africa	2.9 (1.8 to 4.3)	0.77 (0.50 to 1.11)	9.6 (6.1 to 14.1)	2.46 (1.55 to 3.58)	129.09 (57.49 to 233.23)	0.09 (0.07 to 0.11)
	Central Sub-Saharan Africa	1.4 (0.8 to 2)	1.16 (0.72 to 1.70)	3.3 (2.0 to 4.8)	2.65 (1.60 to 3.84)	120.89 (113.15 to 128.91)	−0.01 (−0.01 to −0.01)
	Southern Sub-Saharan Africa	0.7 (0.4 to 1)	0.85 (0.54 to 1.23)	1.8 (1.1 to 2.7)	2.31 (1.45 to 3.39)	99.19 (39.68 to 184.06)	0 (0 to 0.01)

Regions are grouped by GBD super-region and alphabetically ordered.

*ASR* age-standardized rate, *CI* confidence interval, *EAPC* estimated annual percentage change, *GBD* Global Burden of Diseases, Injuries and Risk Factors Study, *SDI* Socio-demographic index, *UI* uncertainty interval.

At the regional level, age-standardized YLD rates were highest in the high-income Asia Pacific region (1.79 [95% UI: 1.15 to 2.63] per 100,000 people) and central Latin America (2.81 [1.78 to 4.05] per 100,000 people) in 2020 and 2021, respectively (Table 1). The lowest age-standardized YLD rates were observed in East Asia for both 2020 (0.19 [95% UI: 0.12 to 0.30] per 100,000 people) and 2021 (0.19 [95% UI: 0.12 to 0.29] per 100,000 people). During the first two years of the COVID-19 pandemic, Central Europe and Eastern Sub-Saharan Africa experienced the most rapid increase in the GBS burden (Supplementary Figs. 2, 3). EAPCs in age-standardized YLD rates were 129.00% (30.68 to 301.28) and 129.09% (57.49 to 233.23), respectively (Table 1). East Asia presented the lowest EAPC in age-standardized YLD rates, which was 0.39% (−2.77 to 3.66).

**Fig. 1:**
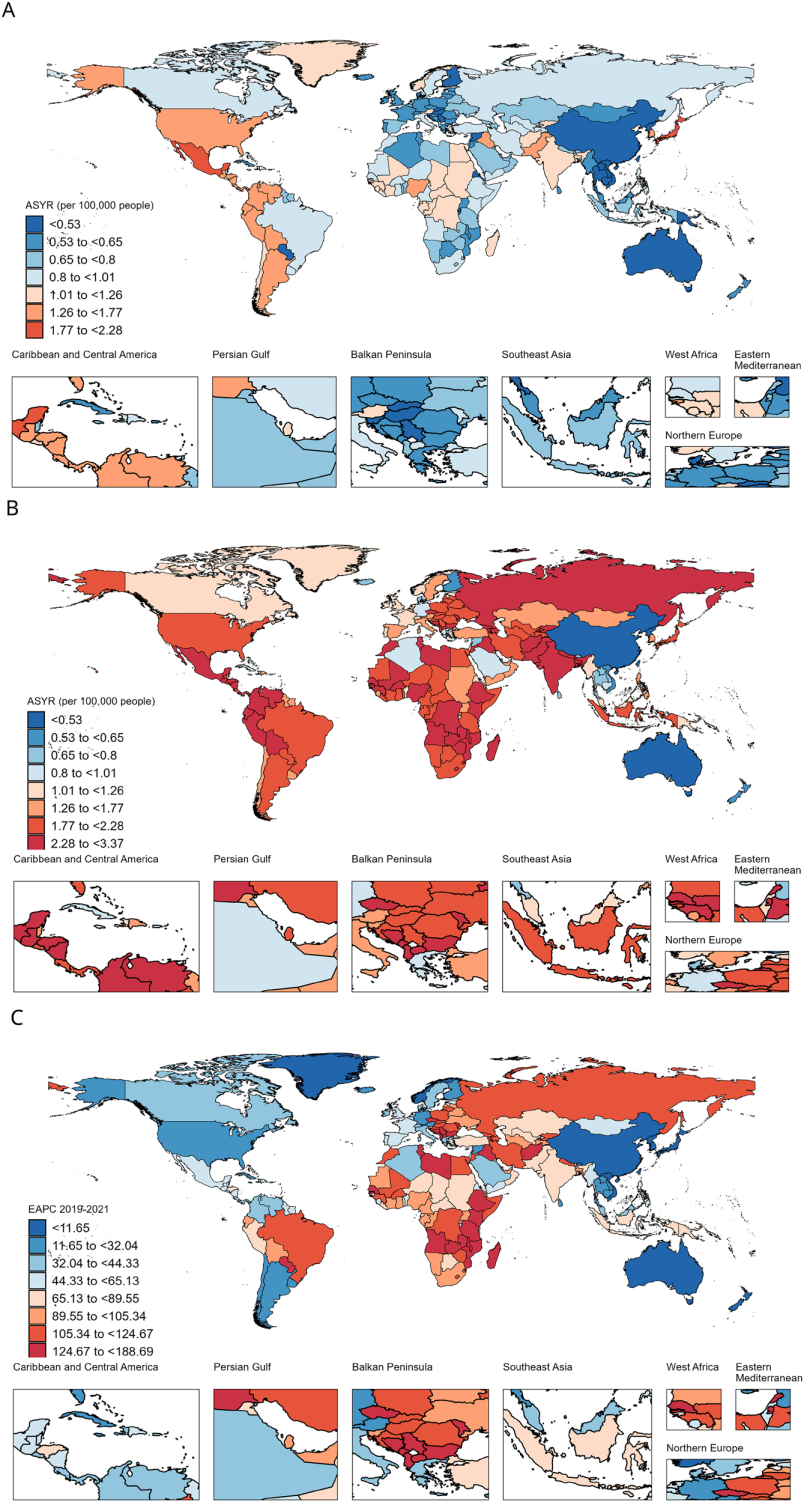
The disability burden of Guillain-Barré syndrome shows a variable spatial distribution between 2019 and 2021. **A** Age-standardized years lived with disability rate (ASYR) in 2020 across 204 countries and territories; **B** ASYR in 2021; **C** Estimated annual percentage changes (EAPC) in ASYR between 2019 and 2021.

Among 204 countries and territories, Singapore, Japan, and Mexico presented the most severe burden of GBS in 2020, while Iraq, the Plurinational State of Bolivia, and North Macedonia had the most severe burden of GBS in 2021 (Fig. 1A, Fig. 1B and Supplementary Table 2). The lowest burden of GBS was observed in East Asian countries and territories, including China, Taiwan (Province of China), and the Democratic People’s Republic of Korea, during the first two years of the COVID-19 pandemic. Fifty-nine out of 204 countries and territories experienced a rapid increase in GBS burden between 2019 and 2021, with EAPCs in age-standardized YLD rates greater than 100% (Fig. 1C and Supplementary Table 2).

### Patterns by sex and age

**Fig. 2:**
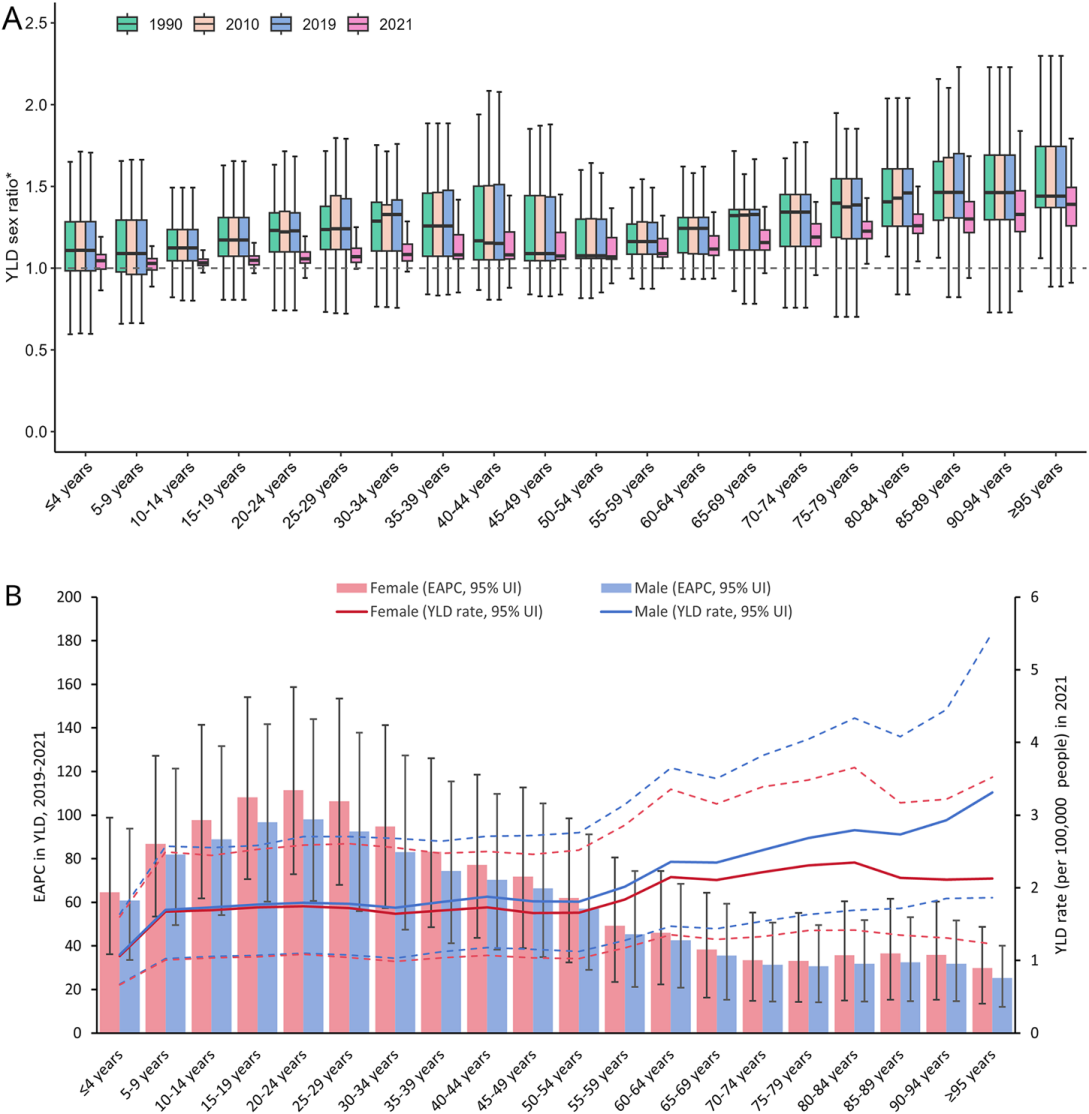
The burden of Guillain-Barré syndrome shows variations in age and sex distribution. **A** Box-and-whisker plots showing the male-to-female ratios of country-level YLD rates across age groups in 1990, 2010, 2019, and 2021 (*n* = 204 countries); **B** Global YLDs rate in 2021 and estimated annual percentage change (EAPC) in YLDs between 2019 and 2021, by age and sex.

All-cause YLD rates of GBS differed between sexes, and the extent of this difference varied across age groups and by geographic locations. In general, male YLD rates were higher than female YLD rates in all age groups, with considerable heterogeneity across countries and territories (Fig. 2A). The greatest variability in the YLD rate male-to-female ratio was observed in the age group of 80 years and older. In 2021, the YLD rate male-to-female ratios were broadly lower than those in 1990, 2010, and 2019, suggesting a narrowing difference in the GBS burden between sexes. Figure 2B shows that all-cause YLD rates of GBS increased more rapidly in females than in males across all age groups during the pandemic, and the COVID-19-specific age-standardized YLD rates were slightly higher in males worldwide and across all regions (Supplementary Table 3).

In 2021, the global burden of GBS was the lowest in children under the age of five and increased with advancing age in both sexes (Fig. 2B and Supplementary Fig. 5). The highest YLD rates were observed in individuals over the age of 94 (2.45 [95% UI: 1.39 to 4.07] per 100,000 people). However, during 2019–2021, YLD rates increased more rapidly in younger age groups, particularly among those aged 15–29 years. The EAPC ranged from 106.31% to 111.45% among females and from 92.53% to 98.03% among males (Fig. 2B).

### Increased GBS burden attributed to COVID-19

**Fig. 3:**
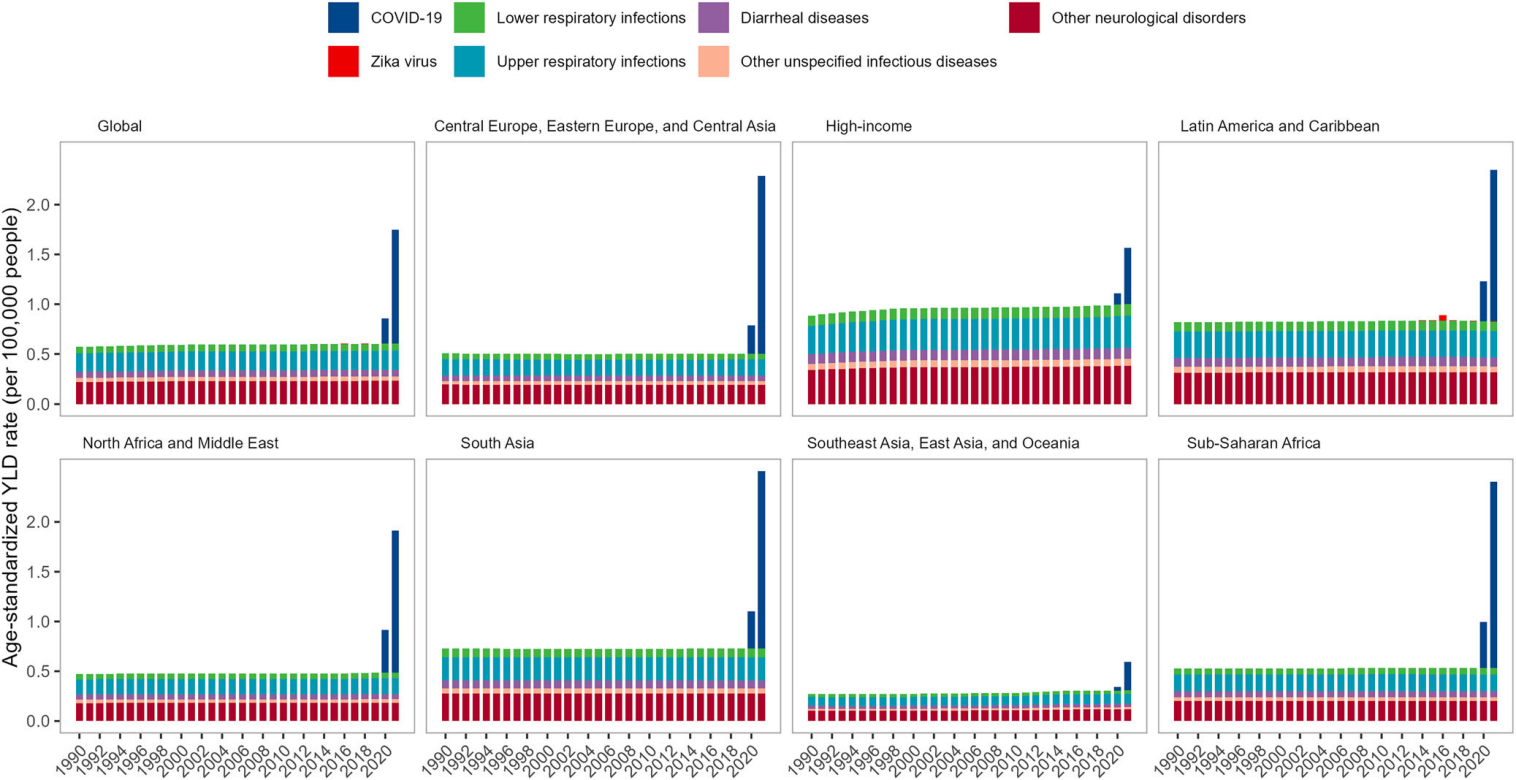
Age-standardized years lived with disability rates of Guillain-Barré syndrome attributed to underlying causes are shown by super regions and years from 1990–2021. Other neurological disorders: idiopathic Guillain-Barré syndrome, the cause is unknown.

At the global level, the YLDs attributable to specific causes of GBS remained stable between 1990 and 2019, with respiratory infections and a residual category labeled as other neurological disorders accounting for over half of the GBS burden (Supplementary Table 4 and Supplementary Fig. 9). In the GBD framework, this category represents idiopathic cases of GBS for which no infectious trigger could be assigned based on available data. During the COVID-19 pandemic, the coronavirus infection was identified as the leading cause associated with the increase in GBS YLDs across all ages, sexes, and GBD regions (Supplementary Figs. 10–13). In 2020, the global cause-specific YLD rates for GBS were 0.25 (95% UI 0.16 to 0.38) per 100,000 people for COVID-19, 0.19 (0.12 to 0.30) per 100,000 people for upper respiratory infections, and 0.23 (0.13 to 0.36) per 100,000 people for other neurological disorders (Fig. 3 and Supplementary Table 5). In 2021, the global cause-specific YLD rates were 1.14 (0.71 to 1.72) per 100,000 people, 0.19 (0.12 to 0.30) per 100,000 people, and 0.23 (0.13 to 0.36) per 100,000 people for COVID-19, upper respiratory infections, and other neurological disorders, respectively. Most regions experienced a higher burden of GBS associated with COVID-19 compared to the global average, except for high-income regions, Southeast Asia, East Asia, and Oceania, which had lower burdens in both 2020 and 2021 (Fig. 3 and Supplementary Table 4).

### Potential influencing factors on GBS burden

Before the COVID-19 pandemic, age-standardized YLD rates were positively associated with SDI levels in high-income regions (Supplementary Fig. 6). Over the period from 2019 to 2021, the EAPC in age-standardized YLD rates showed significant increases across regions, reflecting the global rise in GBS burden (Table 1 and Supplementary Fig. 7). However, this increase was more pronounced in regions with lower SDI levels (Table 1). In 2021, regions with low SDI and low-middle SDI experienced the highest age-standardized YLD rates, which were 2.37 (1.49 to 3.44) per 100,000 people and 2.32 (1.46 to 3.42) per 100,000 people, respectively. During the pandemic, the increase in YLD of GBS was significantly negatively correlated to number of people vaccinated (r_s_ = −0.55), GSI (r_s_ = −0.21), healthcare human resource density (r_s_ = −0.29), healthcare expenditure (r_s_ = −0.27), urbanization rate (r_s_ = −0.18), population density (r_s_ = −0.15) (Supplementary Fig. 15).

### Impact of COVID-19 vaccine coverage on the GBS burden

**Fig. 4:**
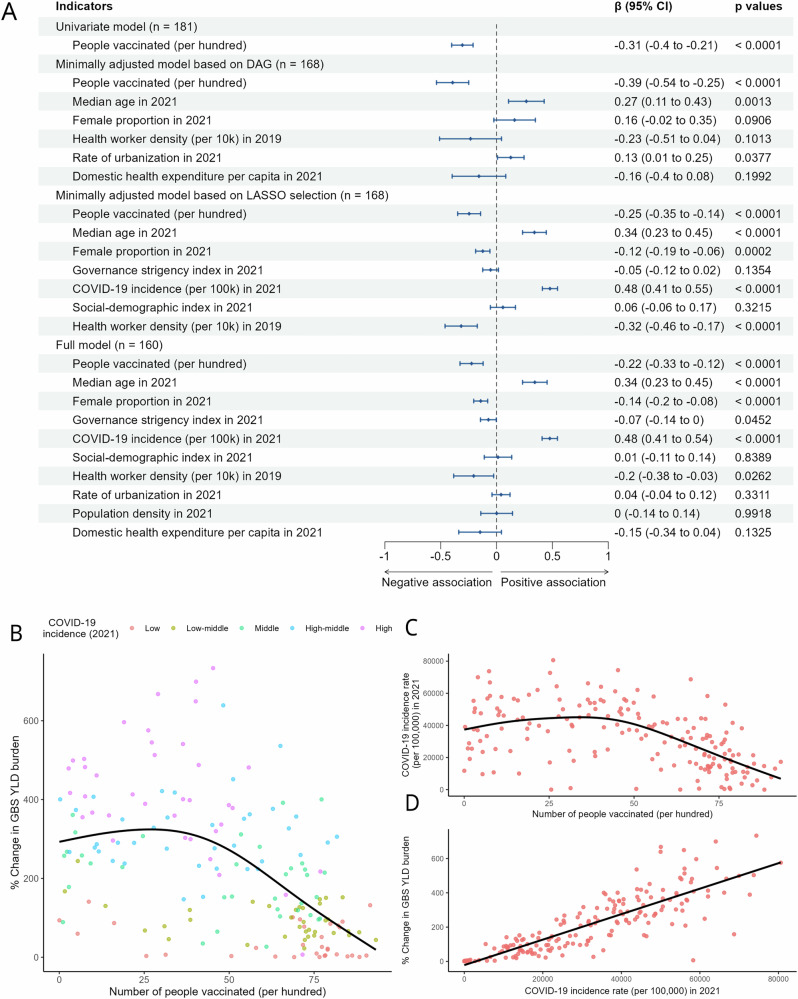
Association between COVID-19 vaccine coverage (number of people vaccinated per hundred) in 2021 and percentage increase in YLD rate of GBS (2021 vs 2019) is assessed through regression modeling and country-level analyses. **A** Regression coefficients derived from generalized linear models with Gaussian distribution and log-link function; **B** relationship between COVID-19 vaccine coverage and increase in GBS burden on country levels; **C** relationship between COVID-19 vaccine coverage and COVID-19 incidence rate on country levels; **D** relationship between COVID-19 incidence rate and increase in GBS burden.

Figure 4A shows the association between the number of people vaccinated (per hundred) and the change in YLD rate of GBS assessed using GLIMs. The univariate analysis revealed a significant negative association (β = −0.31, 95% CI: −0.40 to −0.21, *p* < 0.0001), indicating that higher vaccination rates were associated with a reduction in GBS burden. This inverse association persisted after adjusting for potential confounders in the multivariate models. Specifically, the model based on DAG (β = −0.39, 95% CI: −0.54 to −0.25, *p* < 0.0001), the model based on LASSO selection (β = −0.25, 95% CI: −0.35 to −0.14, *p* < 0.0001), and the full model (β = −0.22, 95% CI: −0.33 to −0.12, *p* < 0.0001) consistently demonstrated a robust and statistically significant association, supporting the hypothesis that increased vaccination coverage is linked to a reduction in GBS burden. Results derived from adjusted GAMs suggest non-linear negative relationships between vaccination coverage rates and age-standardized YLD rates of GBS (Fig. 4B). Stronger associations were observed in countries with moderate vaccination coverage, while weaker associations were found in countries with either lower or higher vaccination coverage.

Furthermore, causal mediation analysis revealed a significant indirect effect of the vaccination coverage on the increase in GBS burden of YLD through the COVID-19 incidence rate. The average causal mediation effect was estimated to be −3.544 (95% CI: −9.275 to −0.820, *p* < 0.001). The average direct effect was significantly negative, with an estimate of −4.402 (95% CI: −7.280 to −2.15, *p* < 0.001). The proportion mediated was estimated to be 0.446 (95% CI: 0.191 to 0.67). Figure 4C and D present a visual representation of the relationships between vaccination coverage and COVID-19 incidence rate, and between COVID-19 incidence rate and the observed increase in the burden of YLD attributed to GBS.

## Discussion

This is the first study that investigated the influence of the COVID-19 pandemic and vaccination on the GBS burden globally. Our findings revealed a substantial increase in the global age-standardized YLDs related to GBS during the first two years of the COVID-19 pandemic, with SARS-CoV-2 infection identified in the GBD estimates as a major associated factor contributing to this rise. We identified significant regional variations, noting that low SDI regions experienced the steepest increase in the GBS burden. Importantly, improving vaccination coverage was the most effective measure to mitigate the post-COVID-19 burden of GBS, despite the existing inequalities in vaccination rates associated with SDI. This study provides a crucial basis for understanding the global epidemiology of GBS during the COVID-19 pandemic and highlights that the benefits of vaccination outweigh the risks in controlling the post-COVID-19 GBS burden.

The association between SARS-CoV-2 infection and GBS has been a subject of debate. Our investigation provides new evidence for this relationship from a global perspective. The observed sharp rise in the burden of GBS during the first two years of the COVID-19 pandemic is consistent with several observational studies^5,6,13^. A self-controlled case series in the UK revealed an incidence rate ratio of 5.25 (95% CI: 3.00 to 9.18) for GBS within 28 days following a positive SARS-CoV-2 test^13^. Similarly, a recent case-control study from Israel reported an odds ratio of 6.30 (95% CI: 2.55 to 15.56) for GBS associated with SARS-CoV-2 infection^14^. SARS-CoV-2 may trigger GBS through both direct nerve invasion and immune dysregulation. The hyperinflammatory “cytokine storm” and blood-nerve barrier disruption appear to promote molecular mimicry, where viral components such as SARS-CoV-2 heat shock protein-like sequences resemble peripheral nerve antigens. This similarity can drive autoantibody production, resulting in complement-mediated nerve damage through demyelination and axonal injury^15^. However, some studies did not observe an increased risk of GBS during the pandemic era, and some even suggested a reduction in GBS incidence compared to previous years^16–18^. A recent meta-analysis revealed an increased risk of GBS in northern Italy, while a slight reduced risk was observed in other countries during the pandemic^19^. The studies included in this meta-analysis primarily involved European countries and only analyzed hospitalized patients^19^, which may lead to potential selection bias. Our study highlights the importance of addressing not only infection control during a pandemic but also the burden of potential post-infectious complications, such as GBS. By recognizing the broader spectrum of pandemic-related health challenges, we can better prepare for and mitigate the multifaceted impacts of future infectious disease outbreaks.

The burden of GBS remained higher in males than in females across all age groups from 2019 to 2021, consistent with the pre-pandemic sex pattern^1^. However, a greater increase in burden was observed in females during this period, which may indicate a change in the sex distribution of GBS during the pandemic. Additionally, the global burden of GBS rose more rapidly among younger age groups of both sexes, particularly those aged 15–29 years. Studies have suggested that immune responses to SARS-CoV-2 infection differ between males and females and across various age groups^20,21^. These differences may contribute to the narrowed disparities between sexes and age groups in GBS following SARS-CoV-2 infection. Further research is needed to validate these changes in sex and age patterns and to explore the underlying causes.

At the regional level, there is a clear variation in the burden of GBS, which is influenced by SDI. Specifically, regions with lower SDI levels experienced more rapid increases in the GBS burden during the first two years of the COVID-19 pandemic. Notably, compared to low SDI countries, high SDI countries experienced faster growth in the GBS burden from 1990 to 2019, as shown in this study and by others^21^. This increase can be attributed to factors such as aging populations, better recognition and diagnostic techniques for GBS, and more healthcare resources^22,23^. However, this trend contrasts with the pandemic period from 2019 to 2021, during which high SDI countries managed to decelerate the growth rate of the GBS burden. Indeed, countries with higher SDI, which typically have more robust healthcare systems and greater financial resources, were better equipped to promptly implement extensive measures to control the spread of SARS-CoV-2. This may have contributed to more effective pandemic management and a relatively smaller increase in GBS burden^24^. In contrast, low SDI countries, with more limited healthcare and economic resources, faced substantial challenges in managing the pandemic. Resource constraints and the limited resilience of their healthcare system may have hindered their ability to control the spread of the virus, potentially contributing to a more pronounced rise in the GBS burden^25^. Therefore, strengthening pandemic preparedness and response mechanisms in low SDI regions is essential to manage infectious disease outbreaks and associated health burdens more effectively. It also highlights the necessity for tailored response strategies that consider regional disparities in healthcare capacity and resource availability.

Vaccination is an effective measure for controlling the transmission of SARS-CoV-2, with estimates suggesting that over 14 million deaths were prevented globally in just the first year of vaccination deployment^7^. Our results revealed a negative correlation between the number of vaccinated individuals and the increase in the burden of GBS, which may be partly explained by reduced COVID-19 incidence. However, significant disparities in vaccination coverage exist between countries. High-income countries have more immediate access to a variety of vaccines and higher vaccination rates, whereas low-income countries face substantial delays in vaccine rollout and consequently worse vaccination effects^26,27^. Research indicates that an additional 45% of COVID-19-related deaths could have been averted if low-income countries had met the 20% vaccination coverage target set by the COVID-19 Vaccines Global Access^7^. Moreover, a one-day delay in the first vaccination in low-income countries was associated with a 1.92% increase in cumulative cases compared to high-income countries^27^. This inequality in vaccination coverage aligns with our findings that vaccination rates were positively correlated with SDI levels. The limited vaccination coverage in low SDI regions was associated with higher COVID-19 transmission and a more pronounced increase in GBS burden. Therefore, enhancing vaccination coverage may help reduce the burden of GBS, possibly through the indirect effect of limiting SARS-CoV-2 transmission. Our results also emphasize the importance of addressing the inequities in vaccine distribution and access, particularly in the low SDI regions. In Africa, for instance, around 56% of population living in rural areas, COVID-19 vaccine distribution faced significant logistical challenges. In addition to inadequate cold-chain capacity, many countries suffer from underdeveloped healthcare infrastructure, resulting in a scarcity of vaccination sites and limited personnel accessibility, thereby contributing to persistently low vaccination coverage (Supplementary Fig. 14)^28^.

There were extensive concerns following the rollout of COVID-19 vaccines, because GBS can be triggered by certain vaccinations^1^. Previous research indicated that adenovirus-vectored vaccines might be associated with an increased risk of GBS^9,13^, while mRNA vaccines were observed to have a lower risk of inducing GBS^9,14^. This difference may be attributed to the immune responses towards the viral vector through molecular mimicry. In contrast, mRNA vaccines lack the viral antigens that could induce such an autoimmune response^29^. However, country-level data on vaccination coverage do not distinguish between vaccine platforms, dosing schedules, or timing of administration—factors that may influence both efficacy and the risk of GBS. Therefore, our findings suggest that higher overall vaccination, regardless of the vaccine type, is associated with a lower burden of GBS at the global level. This association remains after adjusting for SARS-CoV-2 incidence, raising the possibility that vaccination could influence the risk of GBS through additional mechanisms, as observed with other vaccines^30^. Future studies should leverage disaggregated immunization registries to evaluate whether particular COVID-19 vaccine platforms or dosing regimens offer enhanced protection against GBS. Overall, these findings support the favorable risk-benefit profile of COVID-19 vaccination, particularly in relation to its potential role in reducing the GBS burden during the pandemic. Policymakers should advocate for transparent communication about the advantages and potential adverse effects of vaccination, which ensures the public is well-informed and helps in countering vaccine hesitancy.

This study has several limitations. First, the GBS burden analyses were derived from the GBD 2021 framework, which synthesizes data from multiple sources using standardized methods. Although cases were identified using ICD codes (ICD-9: 357.0; ICD-10: G61.0), validation using diagnostic criteria such as NINDS or Brighton criteria was not available. While this coding-based approach is limited in clinical specificity, it remains a widely accepted method for ensuring comparability and scalability in population-level analyses^31–35^. However, diagnostic accuracy may vary substantially across settings, particularly in low-SDI regions where electrodiagnostic capacity and surveillance infrastructure are limited. Mild or atypical GBS cases may go unrecognized, and even severe presentations may be misclassified. For example, although Sub-Saharan Africa’s COVID-19 vaccine coverage is substantially lower than that of Central and Eastern Europe, the smaller apparent increase in GBS burden likely reflects underreporting rather than a true epidemiological decline (Supplementary Fig. 15). Second, the modeling process applied in the GBD framework involves standardized assumptions that carry inherent limitations. The attribution of GBS burden to COVID-19 relied on risk estimates from a large matched analysis of health records, including over one million cases and controls matched on key demographic and clinical variables. While this design offered substantial statistical power to assess rare neurological outcomes such as GBS, the resulting estimates may not fully reflect variation across regions, time periods, or healthcare access. Similarly, a uniform disability weight was applied to all GBS cases, regardless of etiology or setting. These modeling choices, though necessary for comparability and scalability in global burden estimation, may not fully reflect real-world heterogeneity in severity, recovery patterns, or long-term outcomes of GBS. Third, the use of country-level aggregate data introduces the possibility of ecological fallacy, where population-level associations may not apply at the individual level. Although our causal mediation framework estimates the indirect effect of vaccination (via reduced COVID-19 incidence) on GBS burden, the ecological nature of the data inherently limits causal inference. Specifically, we note that individual-level factors such as genetic predisposition, respiratory coinfections, comorbid burden, and regional variation in GBS diagnosis and reporting practices may bias the observed mediator-outcome relationship. Accordingly, our findings should be interpreted with caution and regarded as hypothesis-generating rather than definitive evidence of causality. While special care is required when applying these results to real-world contexts, this study nonetheless provides timely insights into global GBS burden trends during the early pandemic and directions for further research using individual-level data.

In conclusion, the burden of GBS significantly increased during the first two years of the pandemic, in association with the global surge of COVID-19 cases, particularly in those countries with lower SDI levels. More rapid increases in GBS burden were observed in females and younger populations (aged 15–29 years). Despite significant disparities in vaccination coverage between countries, the benefits of vaccination outweighed the risks in managing the post-COVID-19 GBS burden. These insights can support policymakers in planning targeted control measures to manage the burden of diseases that arise as post-infection complications, such as GBS, during future COVID-19 waves or other potential epidemics.

## Methods

### Study design and data sources

We obtained data on the global, regional, and national burden of GBS between 1990 and 2021 from the GBD database (https://vizhub.healthdata.org/gbd-results/)^34^. The primary outcome of the study was age-standardized years lived with disability (YLD) rates and percentage of change in the GBS YLD rate between 2019 and 2021. Prior to the COVID-19 pandemic, the YLD burden of GBS exhibited minimal variation over the preceding 30 years. Therefore, this study employed the percentage of change in the GBS YLD rate between 2019 and 2021 as an indicator of the excess GBS burden attributable to the pandemic. Moreover, the socio-demographic index (SDI)^35^ and Health workers density (measured by number of healthcare worker per 10,000 people)^36^ were downloaded from the GBD database.

We collected data on potential influencing factors for the change in GBS burden during the pandemic, including COVID-19 vaccination coverage in 2021^37^, and the government stringency index (GSI) in both 2020 and 2021^38^, median age (years), female proportion (%), number of healthcare worker (per 10,000), share of urban population (%), per square kilometer population, and per capita total expenditure on health (PPP int. $) from Our World in Data (https://ourworldindata.org/). Secondary analysis of the de-identified data from GBD and OWID did not require additional ethical approval.

### Missing data handling

We extracted vaccination rate data on December 31, 2021, to analyze the potential impact of vaccination against COVID-19 on the GBS burden. However, vaccination rate data might not be available for this day in some countries and territories. Therefore, we filled the data with the nearest values within a window of 1 month before or after the missing point date. Observations (i.e. countries and territories) were excluded from analyses if the missing point could not be filled or the countries and territories could not be found in the OWID database.

### Statistical analyses

To measure the age-standardized YLD rate temporal trends over 1990–2019 and 2019–2021, estimated annual percentage changes (EAPCs) were computed by fitting a linear regression model to the log-transformed age-standardized YLD rate^39^. We additionally performed joinpoint regression models to identify “points” where a significant change in the trend of a data series occurred^40^. Each segment between two adjacent points represented a different linear trend. Weighted Bayesian Information Criterion (WBIC) method was used to select the model with the lowest WBIC, which balanced goodness-of-fit with model complexity^41^.

A matrix of Spearman’s correlation coefficients (rs) and smoothing curves derived from generalized additive models was generated to visualize the pairwise correlations between variables. Univariate and multivariate generalized linear models were applied to assess the association between change in age-standardized YLD rates of GBS from 2019 to 2021 (dependent variable) and number of people vaccinated (primary independent variable). The GLIMs used a Gaussian distribution and log-link function to control for the skewed nature of the dependent variable (Supplementary Figs. 8–19). Residual diagnostics were conducted to assess the adequacy of the GLIM model (Gaussian family with log link). The Shapiro-Wilk test indicated non-normality of residuals (*p* = 0.03). However, as residual normality is not a strict requirement for generalized linear models with a log link, this result does not invalidate the model. Residual plots of deviance and Pearson residuals versus fitted values showed no major patterns or violations (Supplementary Fig. 20). The main model was a minimally adjusted model with adjustment for median age, female proportion, number of healthcare worker, share of urban population, and per capita total expenditure on health, which were selected using a directed acyclic graph (DAG) (Supplementary Fig. 1). Variance inflation factor was used to detect multicollinearity in GLIM models.

To address potential inference concerns and ensure the robustness of results, several sensitivity analyses were performed. We compared parameter estimates across different model specifications and variable selection methods. we fitted generalized linear models using a gamma distribution with a log link and robust standard errors to account for heteroscedasticity and skewed outcome distributions. Then two additional multivariate models were developed: (1) a full model adjusted for median age (years), female proportion (%), GSI, COVID-19 incidence rate (per 100,000), SDI, number of healthcare worker (per 10,000), share of urban population (%), per square kilometer population, and per capita total expenditure on health (PPP int. $) (Supplementary Table 4); (2) a model adjusted for variables based on LASSO selection, including median age, female proportion, GSI, COVID-19 incidence rate, SDI, and number of healthcare worker (per 10,000 people). The independent variables were standardized by centering each variable at its mean and scaling it to have a unit variance prior to model construction.

Furthermore, we employed a mediation analysis to investigate the extent to which the effect of the number of people vaccinated on change in age-standardized YLD rates of GBS from 2019 to 2021 was mediated by COVID-19 incidence rate. Bootstrapping with 1000 simulations was performed to obtain robust estimates of the mediation effect and their 95% confidence intervals (CI). The mediate function from the mediation package in R was utilized.

R (version 4.3.3 and 4.4.0) and Joinpoint Software (version 5.2.0) were used for statistical analysis and data visualization. Two-sided hypothesis tests were conducted with a statistical significance level of 0.05.

## Supplementary information

Supplementary materials_revised - 20250625

## Data Availability

The data used for analyses are publicly available at (1) GBD 2021 database: https://ghdx.healthdata.org/gbd-results-tool. and (2) Our World in Data: https://ourworldindata.org/.
