# Supplementary material for: Impact of COVID-19 vaccination coverage on global disability burden of Guillain-Barré syndrome
Source: NPJ Vaccines. 2025 Aug 2;10:182. doi: 10.1038/s41541-025-01239-1 (PMC12318086; doi:10.1038/s41541-025-01239-1)
Supplement: Supplementary file 1 — Supplementary materials_revised - 20250625 [file 41541_2025_1239_MOESM1_ESM.pdf]

Supplementary Materials

Impact of COVID-19 vaccination coverage on global disability burden of Guillain-Barre syndrome

Table of Contents

Table of Contents 2

1. Supplementary Tables 4

Table 1 Prevalent cases for Guillain-Barré syndrome in 2020 and 2021 and the estimated annual percentage change in the age-standardized rates per 100,000, by GBD region and SDI, from 1990 to 2021 5

Table 2 Years lived with disability of Guillain–Barré syndrome in 2020 and 2021 and the estimated annual percentage change in the age-standardized rates per 100,000 in 204 countries and territories, from 1990 to 2021 7

Table 3 Male and female age-standardized years lived with disability rates (ASYRs) attributed to COVID-19, and ASYR sex ratio, in 2020 and 2021 13

Table 4 Dependent and independent variables in generalized linear models 15

Table 5 Cause-specific age-standardized years lived with disability of Guillain-Barré syndrome at global and supper-regional levels, before and during the years of COVID-19 pandemic 16

Table 6 Sensitivity analysis for the association between COVID-19 vaccination coverage and age-standardized YLD rate at national level in 2021, using generalized linear model with Gaussian distribution and log-link function. 18

Table 7 Sensitivity analysis of parameter estimates across different regression models and variable selection methods 19

2. Supplementary Figures 20

Figure 1 Directed acyclic graph for the association between 20

Figure 2 Joinpoint regression analysis of the age-standardized years lived with disability rate of Guillain-Barré syndrome by seven GBD super regions, 1990–2021. 21

Figure 3 Joinpoint regression analysis of the age-standardized years lived with disability rate of Guillain-Barré syndrome by High-income regions, 1990–2021. 22

Figure 4 Joinpoint regression analysis of the age-standardized years lived with disability rate of Guillain-Barré syndrome by Latin America regions, 1990–2021. 23

Figure 5 Age-sex distribution of prevalence of Guillain-Barré syndrome in 2021 24

Figure 6 Age-standardized years lived with disability rates (YLDs) of Guillain-Barré syndrome versus Socio-demographic Index, globally and across 21 regions. Each point shows the observed age-standardized YLDs rate for each region from 1990 to 2019. 25

Figure 7 Joinpoint regression analysis of the age-standardized years lived with disability rate of Guillain-Barré syndrome globally and by quintile of the socio-demographic index, 1990–2021. 26

Figure 8 Association between estimated annual percentage change in age-standardized years lived with disability rate during the COVID-19 pandemic, across 204 countries and territories. 27

Figure 9 Age-standardized years lived with disability rates of Guillain-Barré syndrome attributed to causes excluding COVID-19, by super region and years from 1990-2021. 28

Notes: Other neurological disorders: idiopathic Guillain-Barré syndrome, the cause is unknown. 28

Figure 10 Age-standardized years lived with disability rates of Guillain-Barré syndrome attributed to underlying causes in females, by super region and years from 1990-2021. 29

Notes: Other neurological disorders: idiopathic Guillain-Barré syndrome, the cause is unknown. 29

Figure 11 Age-standardized years lived with disability rates of Guillain-Barré syndrome attributed to underlying causes in males, by super region and years from 1990-2021. 30

Notes: Other neurological disorders: idiopathic Guillain-Barré syndrome, the cause is unknown. 30

Figure 12 Years lived with disability rates of Guillain-Barré syndrome attributed to specific causes by age group in 2020. 31

Notes: Other neurological disorders: idiopathic Guillain-Barré syndrome, the cause is unknown. 31

Figure 13 Years lived with disability rates of Guillain-Barré syndrome attributed to specific causes by age group in 2021. 32

Notes: Other neurological disorders: idiopathic Guillain-Barré syndrome, the cause is unknown. 32

Figure 14 Number of people vaccinated per hundred across 181 countries and territories, by 31.12.2021. 33

The Number of people vaccinated per hundred may be higher than 100% of population. Because changes in population due to migration, birth or death may not be accurately reflected in latest population estimates. If a country has seen increasing migration trends or if a national population statistic is lower than the count recorded in statistical calculations, it can result in a vaccination coverage percentage greater than 100%. 33

Figure 15 Geographic heterogeneity in the relationship between COVID-19 vaccination coverage and GBS YLD change across 181 countries and territories, by 31.12.2021. 34
Figure 16 Age-standardized COVID-19 incidence by years and socio-demographic index 35
Figure 17 Spearman correlation matrix across disease burden of GBS and potential influencing factors. 36
Figure 18 Histogram of percentage change in GBS YLD burden without transformation. 37
Figure 19 Q-Q plot of percentage change in GBS YLD burden without transformation. 37
Figure 20 Residuals versus fits plot for the GLIM model with Gaussian distribution and log-link function 38
Note: Shapiro-Wilk test of residual normality (W = 0.98226, *p* = 0.0304). 38

1. Supplementary Tables

Supplementary Table 1 Prevalent cases for Guillain-Barré syndrome in 2020 and 2021 and the estimated annual percentage change in the age-standardized rates per 100,000, by GBD region and SDI, from 1990 to 2021

GBD supper regions	GBD regions	2020		2021		2019–2021	1990–2019
		Count (thousands)	ASR of prevalence (per 100,000 people)	Count (thousands)	ASR of prevalence (per 100,000 people)	EAPC	EAPC
Global		230.1 (194.3 to 269.7)	2.89 (2.45 to 3.4)	471.9 (389.2 to 554.1)	5.91 (4.87 to 6.97)	70.35 (38.73 to 109.17)	0.16 (0.14 to 0.18)
Low SDI		33.3 (27.3 to 39.9)	3.39 (2.79 to 4.03)	82.4 (65.1 to 99.3)	8.03 (6.39 to 9.6)	103.38 (71.09 to 141.77)	-0.01 (-0.02 to 0)
Low-middle SDI		64.4 (53.4 to 77.4)	3.54 (2.94 to 4.21)	147.3 (118.9 to 177.6)	7.84 (6.36 to 9.36)	88.25 (56.64 to 126.25)	0.02 (0.01 to 0.03)
Middle SDI		60.7 (50.8 to 71.7)	2.46 (2.07 to 2.93)	124.5 (102.5 to 147)	5.02 (4.11 to 5.98)	68.69 (36.08 to 109.13)	0.42 (0.4 to 0.44)
High-middle SDI		25.5 (21.2 to 30.5)	1.79 (1.49 to 2.15)	53.5 (44.4 to 62.3)	3.82 (3.16 to 4.56)	67.04 (26.52 to 120.54)	0.11 (0.09 to 0.14)
High SDI		46 (39.2 to 53.5)	3.45 (2.95 to 4.04)	63.8 (55.4 to 73.6)	4.99 (4.29 to 5.78)	28.02 (11.45 to 47.05)	0.11 (0.06 to 0.16)
Central Europe, eastern Europe, and central Asia		12 (10 to 14.5)	2.65 (2.19 to 3.17)	35 (28.7 to 42)	7.74 (6.31 to 9.33)	113.4 (49.75 to 204.11)	-0.01 (-0.02 to 0)
	Central Asia	2.8 (2.2 to 3.4)	3.04 (2.43 to 3.65)	6.6 (5.1 to 8.3)	6.98 (5.39 to 8.72)	94.95 (61.83 to 134.84)	0.01 (0 to 0.01)
	Central Europe	2.5 (2.1 to 3.1)	1.92 (1.61 to 2.31)	9.3 (7.6 to 11)	7.24 (5.83 to 8.66)	129.13 (30.68 to 301.74)	-0.14 (-0.18 to -0.1)
	Eastern Europe	6.7 (5.5 to 8.1)	2.89 (2.39 to 3.46)	19.1 (15.4 to 23.1)	8.43 (6.75 to 10.21)	114.03 (51.08 to 203.22)	0.01 (0.01 to 0.01)
High-income		50.1 (42.8 to 58.5)	3.75 (3.21 to 4.39)	67.8 (58.8 to 78)	5.29 (4.56 to 6.13)	25.98 (10.67 to 43.42)	0.28 (0.22 to 0.33)
	Australasia	0.6 (0.4 to 0.7)	1.59 (1.28 to 1.97)	0.6 (0.5 to 0.7)	1.62 (1.31 to 2.00)	1.52 (1.01 to 2.04)	1.24 (1.02 to 1.46)
	High-income Asia Pacific	11 (9.2 to 13.2)	6.03 (4.97 to 7.40)	11.6 (9.8 to 13.8)	6.34 (5.28 to 7.74)	2.84 (0.31 to 5.43)	0.15 (0.1 to 0.2)
	High-income North America	22.6 (19.6 to 26.3)	4.67 (4.05 to 5.40)	31.5 (27.5 to 35.8)	6.93 (5.96 to 7.90)	30.43 (12.72 to 50.92)	0.45 (0.33 to 0.57)
	Southern Latin America	3 (2.5 to 3.6)	4.23 (3.58 to 5.09)	4.3 (3.6 to 5.2)	6.05 (5.02 to 7.31)	25.64 (8.46 to 45.55)	0.24 (0.16 to 0.32)
	Western Europe	12.9 (10.8 to 15.3)	2.33 (1.97 to 2.74)	19.8 (16.9 to 23.1)	3.81 (3.21 to 4.46)	42.25 (21.44 to 66.63)	0.71 (0.66 to 0.76)
Latin America and Caribbean		24.6 (21 to 28.7)	4.15 (3.56 to 4.86)	47.7 (39.5 to 56.5)	7.93 (6.57 to 9.44)	67.99 (45.29 to 94.25)	0.11 (0.06 to 0.16)
	Andean Latin America	3 (2.5 to 3.6)	4.76 (3.96 to 5.61)	5.9 (4.6 to 7.3)	8.94 (7.05 to 11.05)	86.76 (85.6 to 87.93)	-0.17 (-0.21 to -0.13)
	Caribbean	1.2 (1 to 1.5)	2.58 (2.06 to 3.15)	1.9 (1.5 to 2.3)	3.88 (3.03 to 4.82)	36.61 (22.32 to 52.59)	0.02 (-0.1 to 0.15)
	Tropical Latin America	6.4 (5.4 to 7.5)	2.71 (2.27 to 3.18)	16 (13 to 19.4)	6.76 (5.48 to 8.21)	124.12 (98.15 to 153.5)	0.04 (-0.04 to 0.12)
	Central Latin America	13.9 (11.8 to 16.4)	5.60 (4.75 to 6.63)	24 (20.1 to 28.1)	9.49 (7.97 to 11.13)	48.25 (27.37 to 72.55)	0.07 (0.03 to 0.1)
North Africa and Middle East	North Africa and Middle East	18.1 (14.4 to 22.4)	3.09 (2.47 to 3.77)	38.9 (30.7 to 47.9)	6.47 (5.13 to 7.94)	98.72 (87.25 to 110.9)	0.04 (0.02 to 0.06)
South Asia	South Asia	66.1 (55.6 to 79.1)	3.73 (3.14 to 4.44)	155.1 (126.7 to 185.3)	8.48 (6.93 to 10.11)	85.92 (47.92 to 133.67)	0 (0 to 0)
Southeast Asia, east Asia, and Oceania		25.6 (20.1 to 31.7)	1.15 (0.92 to 1.44)	43.2 (35.7 to 51.5)	2.01 (1.66 to 2.40)	39.62 (8 to 80.49)	0.44 (0.37 to 0.52)
	Southeast Asia	15.4 (12.4 to 18.9)	2.22 (1.80 to 2.71)	32.9 (27.2 to 39)	4.69 (3.89 to 5.55)	55.91 (10.42 to 120.12)	0.09 (0.07 to 0.11)

Supplementary Table 1 Prevalent cases for Guillain-Barré syndrome in 2020 and 2021 and the estimated annual percentage change in the age-standardized rates per 100,000, by GBD region and SDI, from 1990 to 2021

GBD supper regions	GBD regions	2020 Count (thousands)	2020 ASR of prevalence (per 100,000 people)	2021 Count (thousands)	2021 ASR of prevalence (per 100,000 people)	2019–2021 EAPC	1990–2019 EAPC
	East Asia	10 (7.5 to 12.9)	0.64 (0.50 to 0.83)	9.9 (7.4 to 12.9)	0.63 (0.48 to 0.82)	0.39 (-2.78 to 3.66)	0.32 (0.13 to 0.5)
	Oceania	0.2 (0.1 to 0.2)	1.31 (1.03 to 1.65)	0.4 (0.3 to 0.6)	3.21 (2.22 to 4.54)	59.32 (-1.75 to 158.37)	0 (0 to 0)
Sub-Saharan Africa		33.5 (27.4 to 40.5)	3.36 (2.77 to 3.99)	84.1 (66.9 to 101.4)	8.13 (6.56 to 9.72)	112.86 (84.03 to 146.21)	0.02 (0.01 to 0.02)
	Western Sub-Saharan Africa	16.8 (13.6 to 20.3)	3.96 (3.28 to 4.69)	34.2 (27 to 41.3)	7.79 (6.18 to 9.38)	100.98 (96.14 to 105.94)	-0.01 (-0.02 to -0.01)
	Eastern Sub-Saharan Africa	9.8 (8 to 12.1)	2.60 (2.14 to 3.12)	32.6 (26.1 to 39.4)	8.33 (6.78 to 10.04)	129.58 (57.49 to 234.67)	0.09 (0.07 to 0.11)
	Central Sub-Saharan Africa	4.7 (3.6 to 5.8)	3.92 (3.00 to 4.80)	11.1 (7.9 to 13.9)	8.97 (6.38 to 11.12)	121.29 (113.26 to 129.62)	-0.01 (-0.01 to -0.01)
	Southern Sub-Saharan Africa	2.2 (1.8 to 2.7)	2.87 (2.37 to 3.46)	6.2 (4.9 to 7.5)	7.82 (6.24 to 9.42)	99.29 (39.74 to 184.23)	0 (0 to 0.01)

Regions are grouped by GBD super-region and alphabetically ordered. ASR=age-standardized rate. CI=confidence interval. EAPC=estimated annual percentage change. GBD=Global Burden of Diseases, Injuries, and Risk Factors Study. SDI=Socio-demographic index. UI=uncertainty interval.

Supplementary Table 2 Years lived with disability of Guillain–Barré syndrome in 2020 and 2021 and the estimated annual percentage change in the age-standardized rates per 100,000 in 204 countries and territories, from 1990 to 2021

Country and territory	ASR in 2020 (per 100,000)	ASR in 2021 (per 100,000)	2019-2021 EAPC in ASR	1990-2019 EAPC in ASR
Afghanistan	1.1 (0.7 to 1.7)	2.4 (1.2 to 3.8)	125.19 (112.92 to 138.15)	0 (-0.01 to 0)
Albania	0.8 (0.5 to 1.2)	3.2 (1.8 to 4.8)	173.72 (78.89 to 318.8)	0 (0 to 0)
Algeria	0.6 (0.4 to 0.9)	0.9 (0.5 to 1.4)	36.64 (29.23 to 44.49)	0 (0 to 0)
American Samoa	0.4 (0.2 to 0.6)	0.4 (0.3 to 0.7)	9.34 (-0.11 to 19.67)	0.1 (0 to 0.2)
Andorra	0.9 (0.6 to 1.4)	1.8 (1.1 to 2.6)	83.86 (73.48 to 94.86)	-0.02 (-0.03 to -0.02)
Angola	0.9 (0.5 to 1.3)	2.8 (1.4 to 4.2)	126.52 (52.64 to 236.15)	-0.03 (-0.03 to -0.03)
Antigua and Barbuda	0.7 (0.4 to 1)	1 (0.6 to 1.4)	23.35 (2.55 to 48.36)	0.22 (-0.03 to 0.48)
Argentina	1.4 (0.9 to 2)	2 (1.2 to 2.9)	24.91 (7.34 to 45.35)	0.2 (0.09 to 0.31)
Armenia	1.1 (0.7 to 1.6)	2.8 (1.6 to 4.2)	127.58 (96.16 to 164.03)	0.01 (0.01 to 0.01)
Australia	0.4 (0.3 to 0.7)	0.4 (0.3 to 0.7)	1.88 (1.2 to 2.57)	1.76 (1.45 to 2.06)
Austria	1.1 (0.8 to 1.6)	1.7 (1 to 2.4)	24.49 (4.54 to 48.25)	1.2 (1 to 1.41)
Azerbaijan	0.8 (0.5 to 1.2)	2.6 (1.4 to 4.2)	120.43 (38.68 to 250.37)	0.02 (0.02 to 0.03)
Bahamas	0.7 (0.5 to 1.1)	1.2 (0.7 to 1.9)	39.63 (15.98 to 68.11)	0.05 (0.01 to 0.08)
Bahrain	0.8 (0.5 to 1.1)	1.9 (1.1 to 2.9)	101.44 (55.26 to 161.36)	0.01 (0.01 to 0.01)
Bangladesh	1.1 (0.7 to 1.7)	2.5 (1.4 to 3.8)	94 (64.24 to 129.16)	0 (0 to 0)
Barbados	0.6 (0.4 to 0.9)	0.8 (0.5 to 1.2)	14.55 (0.83 to 30.14)	0.09 (-0.01 to 0.19)
Belarus	0.8 (0.5 to 1.2)	1.9 (0.9 to 3.4)	95.75 (53.3 to 149.97)	0 (0 to 0.01)
Belgium	0.8 (0.5 to 1.1)	1.3 (0.8 to 1.9)	57.19 (44.18 to 71.38)	0.01 (0.01 to 0.01)
Belize	0.7 (0.5 to 1.1)	1.5 (0.8 to 2.5)	55.17 (11.69 to 115.57)	0.22 (0.02 to 0.41)
Benin	0.7 (0.4 to 1.1)	1.2 (0.7 to 2.1)	51.73 (27.75 to 80.21)	0 (-0.01 to 0)
Bermuda	0.7 (0.4 to 1)	0.9 (0.6 to 1.3)	20 (3.78 to 38.74)	0.01 (0.01 to 0.01)
Bhutan	0.7 (0.4 to 1)	0.7 (0.5 to 1.1)	4.64 (1.02 to 8.4)	-0.01 (-0.01 to -0.01)
Bolivia (Plurinational State of)	1.6 (1 to 2.4)	3.3 (2.1 to 4.9)	104.91 (101.93 to 107.94)	0.02 (0.02 to 0.03)
Bosnia and Herzegovina	0.6 (0.4 to 0.9)	2.6 (1.5 to 3.9)	177.06 (71.06 to 348.74)	-0.25 (-0.39 to -0.1)
Botswana	0.6 (0.4 to 0.9)	1.9 (1 to 3.1)	86.76 (-3.52 to 261.53)	0 (0 to 0.01)
Brazil	0.8 (0.5 to 1.2)	2 (1.3 to 2.9)	123.7 (100.57 to 149.51)	0.04 (-0.04 to 0.12)
Brunei Darussalam	1.7 (1.1 to 2.6)	1.8 (1.1 to 2.7)	2.15 (0.5 to 3.84)	-0.01 (-0.02 to 0)
Bulgaria	0.6 (0.4 to 0.9)	2.6 (1.3 to 4.1)	147.58 (31.63 to 365.68)	0 (-0.01 to 0)
Burkina Faso	1 (0.6 to 1.6)	2.6 (1.5 to 3.9)	117.65 (81.37 to 161.19)	0 (0 to 0)
Burundi	0.5 (0.3 to 0.7)	0.9 (0.6 to 1.3)	39.16 (6.52 to 81.79)	0.03 (0.02 to 0.03)
Cabo Verde	0.9 (0.5 to 1.4)	2.2 (1.2 to 3.6)	102.9 (63.26 to 152.16)	0.14 (-0.04 to 0.33)
Cambodia	0.5 (0.3 to 0.8)	0.9 (0.5 to 1.3)	28.91 (-3.08 to 71.47)	0.01 (0.01 to 0.01)
Cameroon	0.9 (0.5 to 1.5)	2 (0.6 to 3.5)	91.57 (72.18 to 113.14)	0 (0 to 0)
Canada	0.9 (0.6 to 1.4)	1.2 (0.8 to 1.7)	32.57 (29.05 to 36.19)	0.84 (0.68 to 0.99)
Central African Republic	1.2 (0.7 to 2.1)	1.8 (1.1 to 3)	83.87 (42.06 to 137.99)	-0.01 (-0.01 to -0.01)
Chad	1.2 (0.7 to 2.2)	2 (1.1 to 3.4)	89.07 (56.56 to 128.33)	0.03 (0.03 to 0.03)
Chile	1 (0.7 to 1.4)	1.4 (0.9 to 2.1)	27.78	0.58

Supplementary Table 2 Years lived with disability of Guillain–Barré syndrome in 2020 and 2021 and the estimated annual percentage change in the age-standardized rates per 100,000 in 204 countries and territories, from 1990 to 2021

Country and territory	ASR in 2020 (per 100,000)	ASR in 2021 (per 100,000)	2019-2021 EAPC in ASR	1990-2019 EAPC in ASR
			(14.59 to 42.49)	(0.49 to 0.67)
China	0.2 (0.1 to 0.3)	0.2 (0.1 to 0.3)	0.33 (-3.03 to 3.79)	0.31 (0.11 to 0.52)
Colombia	1.4 (0.9 to 2)	2.4 (1.5 to 3.5)	43.8 (15.69 to 78.74)	0.05 (-0.02 to 0.11)
Comoros	0.6 (0.4 to 1)	2.6 (1.6 to 3.8)	141.35 (28.03 to 354.94)	0 (0 to 0)
Congo	1.1 (0.7 to 1.6)	2.1 (1.3 to 3)	94.46 (94.03 to 94.9)	0.02 (0.01 to 0.02)
Cook Islands	0.4 (0.3 to 0.6)	0.7 (0.4 to 1)	36.39 (12.7 to 65.06)	-0.01 (-0.01 to -0.01)
Costa Rica	1.3 (0.8 to 1.9)	2.3 (1.4 to 3.4)	39.96 (9.69 to 78.6)	0 (-0.02 to 0.03)
Côte d'Ivoire	1.2 (0.7 to 1.8)	2.1 (1.2 to 3.2)	96.95 (77.23 to 118.86)	0 (0 to 0)
Croatia	0.5 (0.3 to 0.8)	1.9 (1.2 to 2.9)	115.08 (19.54 to 286.95)	0.02 (0.02 to 0.02)
Cuba	0.6 (0.4 to 0.9)	0.9 (0.6 to 1.3)	19.98 (-1.29 to 45.85)	0.07 (0.01 to 0.13)
Cyprus	0.5 (0.3 to 0.8)	0.9 (0.6 to 1.2)	28.55 (0.46 to 64.49)	0 (-0.01 to 0)
Czechia	0.6 (0.4 to 0.9)	2.7 (1.6 to 4)	152.23 (34.42 to 373.27)	0.02 (0.02 to 0.02)
Democratic People's Republic of Korea	0.2 (0.2 to 0.4)	0.2 (0.2 to 0.4)	1.1 (0.79 to 1.41)	0.01 (0.01 to 0.01)
Democratic Republic of the Congo	1.3 (0.7 to 1.9)	2.7 (1.6 to 4)	123.73 (115.05 to 132.76)	-0.01 (-0.01 to -0.01)
Denmark	0.5 (0.3 to 0.7)	0.7 (0.5 to 1.1)	35.96 (14.86 to 60.94)	1.43 (1.03 to 1.82)
Djibouti	1 (0.6 to 1.6)	1.8 (1 to 2.8)	96.64 (73.3 to 123.11)	0.02 (0.02 to 0.02)
Dominica	0.6 (0.4 to 1)	0.9 (0.5 to 1.2)	16.16 (0.07 to 34.84)	0.4 (-0.09 to 0.88)
Dominican Republic	0.9 (0.6 to 1.4)	1.4 (0.8 to 2.3)	51.61 (45.93 to 57.51)	0.11 (-0.03 to 0.24)
Ecuador	1.3 (0.8 to 1.9)	2.5 (1.5 to 3.8)	97.74 (97.64 to 97.83)	-0.79 (-0.89 to -0.68)
Egypt	1.2 (0.5 to 1.9)	2.2 (0.7 to 3.6)	113.19 (81.67 to 150.17)	0 (0 to 0)
El Salvador	1.2 (0.8 to 1.8)	1.9 (1.1 to 2.8)	33.07 (13.34 to 56.23)	0.23 (0.12 to 0.34)
Equatorial Guinea	1.6 (1 to 2.4)	1.9 (1.1 to 2.9)	87.68 (15.75 to 204.29)	0.04 (0.04 to 0.05)
Eritrea	0.5 (0.3 to 0.7)	1 (0.6 to 1.5)	50.3 (0.64 to 124.45)	0.01 (0.01 to 0.01)
Estonia	0.5 (0.3 to 0.8)	1.2 (0.8 to 2)	57.73 (1.07 to 146.13)	0.01 (0.01 to 0.01)
Eswatini	0.8 (0.5 to 1.1)	2.3 (1.2 to 4)	107.05 (38.15 to 210.3)	0 (0 to 0)
Ethiopia	0.9 (0.6 to 1.3)	2.9 (1.8 to 4.3)	141.33 (67.51 to 247.69)	0.01 (0 to 0.01)
Fiji	0.4 (0.2 to 0.6)	0.9 (0.5 to 1.4)	53.92 (-3.89 to 146.48)	0 (-0.01 to 0)
Finland	0.4 (0.3 to 0.6)	0.5 (0.4 to 0.8)	23 (11.03 to 36.26)	0.46 (0.38 to 0.54)
France	0.6 (0.4 to 0.8)	1 (0.6 to 1.5)	58.34 (39.52 to 79.71)	-0.39 (-0.6 to -0.17)
Gabon	1.1 (0.6 to 1.8)	2 (0.9 to 3.2)	91.94 (76.17 to 109.11)	-0.01 (-0.01 to -0.01)
Gambia	1.1 (0.7 to 1.6)	2.5 (1.4 to 4)	116.08 (91.17 to 144.24)	-0.02 (-0.02 to -0.01)
Georgia	0.6 (0.4 to 0.9)	2.7 (1.2 to 4.2)	123.81 (13.07 to 343)	-0.14 (-0.18 to -0.1)
Germany	0.6 (0.4 to 0.9)	1 (0.6 to 1.4)	28.18 (5.19 to 56.19)	0.48 (0.32 to 0.64)
Ghana	1 (0.6 to 1.4)	2.1 (1.2 to 3.2)	97.6 (74.32 to 123.99)	-0.02 (-0.02 to -0.02)
Greece	0.5 (0.3 to 0.8)	1 (0.6 to 1.4)	37.68 (0.83 to 87.99)	1.59 (1.24 to 1.94)
Greenland	1 (0.6 to 1.5)	1.1 (0.7 to 1.6)	3.26 (0.46 to 6.14)	0.04 (0.04 to 0.04)
Grenada	0.6 (0.4 to 0.9)	0.9 (0.6 to 1.3)	19.48 (-1.24 to 44.54)	0.13 (-0.03 to 0.29)

Supplementary Table 2 Years lived with disability of Guillain–Barré syndrome in 2020 and 2021 and the estimated annual percentage change in the age-standardized rates per 100,000 in 204 countries and territories, from 1990 to 2021

Country and territory	ASR in 2020 (per 100,000)	ASR in 2021 (per 100,000)	2019-2021 EAPC in ASR	1990-2019 EAPC in ASR
Guam	0.6 (0.4 to 0.9)	1.4 (0.8 to 2.3)	96.5 (63.32 to 136.42)	-0.01 (-0.01 to -0.01)
Guatemala	1.6 (1 to 2.3)	2.8 (1.7 to 4.3)	57.17 (36.93 to 80.39)	0.08 (-0.04 to 0.2)
Guinea	1.2 (0.8 to 1.9)	2.5 (1.5 to 3.9)	114.44 (102.3 to 127.32)	0 (0 to 0)
Guinea-Bissau	1.4 (0.8 to 2.3)	1.8 (1 to 2.9)	81.29 (18.94 to 176.33)	-0.01 (-0.01 to -0.01)
Guyana	0.8 (0.5 to 1.1)	1.6 (0.8 to 2.6)	59.38 (16.64 to 117.79)	0 (-0.01 to 0.01)
Haiti	0.9 (0.5 to 1.3)	1.2 (0.6 to 1.9)	37.48 (36.12 to 38.85)	0.02 (0 to 0.04)
Honduras	1.5 (1 to 2.3)	3.2 (2 to 4.8)	75.71 (41.76 to 117.8)	0.18 (0.03 to 0.33)
Hungary	0.5 (0.3 to 0.8)	2 (1.2 to 3.1)	118.3 (16.44 to 309.24)	0 (0 to 0.01)
Iceland	0.6 (0.4 to 0.9)	0.7 (0.4 to 1)	12.32 (9.7 to 15)	0.01 (0.01 to 0.01)
India	1.1 (0.7 to 1.6)	2.5 (1.6 to 3.7)	85.26 (42.45 to 140.95)	0.01 (0 to 0.01)
Indonesia	0.7 (0.5 to 1.1)	1.9 (1.2 to 2.7)	79.94 (16.45 to 178.05)	0 (0 to 0)
Iran (Islamic Republic of)	0.8 (0.5 to 1.2)	2 (1.2 to 2.8)	106.62 (74.81 to 144.23)	0.19 (0.08 to 0.3)
Iraq	1.3 (0.8 to 2)	3.3 (1.8 to 5)	163.88 (145.54 to 183.61)	0 (0 to 0)
Ireland	0.6 (0.4 to 0.9)	1 (0.6 to 1.5)	39.08 (21.48 to 59.23)	0 (0 to 0.01)
Israel	0.6 (0.4 to 0.9)	1.1 (0.7 to 1.7)	48.42 (18.31 to 86.21)	0.01 (0.01 to 0.01)
Italy	0.8 (0.5 to 1.2)	1.3 (0.8 to 1.8)	34.33 (16.59 to 54.78)	0.24 (0.22 to 0.27)
Jamaica	0.7 (0.4 to 1)	1.1 (0.6 to 1.6)	29.55 (3.31 to 62.45)	0.4 (-0.1 to 0.91)
Japan	1.8 (1.2 to 2.7)	1.9 (1.3 to 2.8)	3.59 (0.21 to 7.07)	0.21 (0.14 to 0.28)
Jordan	0.6 (0.4 to 0.9)	2.4 (1.3 to 3.7)	123.26 (16.43 to 328.12)	0 (0 to 0)
Kazakhstan	0.8 (0.5 to 1.2)	1.8 (0.9 to 3.1)	80.2 (42.59 to 127.74)	0.01 (0.01 to 0.01)
Kenya	1 (0.6 to 1.4)	2.9 (1.9 to 4.3)	136.92 (79.31 to 213.04)	0.01 (-0.01 to 0.02)
Kiribati	0.4 (0.2 to 0.6)	0.4 (0.2 to 0.6)	1.46 (-0.19 to 3.14)	0 (0 to 0)
Kuwait	0.8 (0.5 to 1.2)	1.6 (0.9 to 2.7)	77.38 (53.59 to 104.85)	0.03 (0.02 to 0.04)
Kyrgyzstan	1.2 (0.7 to 1.8)	2.4 (1.4 to 3.9)	112.05 (101.11 to 123.59)	0.01 (0.01 to 0.02)
Lao People's Democratic Republic	0.5 (0.3 to 0.8)	0.7 (0.4 to 1)	12.55 (-1.28 to 28.32)	0.01 (0.01 to 0.01)
Latvia	0.5 (0.3 to 0.8)	1.8 (0.9 to 3.5)	89.59 (2.48 to 250.75)	0.01 (0.01 to 0.01)
Lebanon	0.7 (0.4 to 1.3)	2.8 (1.6 to 4.6)	142.42 (40.32 to 318.82)	0 (0 to 0)
Lesotho	0.7 (0.4 to 1)	2.5 (1.3 to 4.3)	114.31 (20.23 to 281.99)	0.02 (0.02 to 0.03)
Liberia	1.2 (0.7 to 1.8)	1.8 (1.1 to 2.8)	83.8 (53.01 to 120.77)	0.01 (0 to 0.01)
Libya	0.7 (0.4 to 1.1)	2.8 (1.5 to 4.2)	153.95 (59.07 to 305.41)	0.74 (0.6 to 0.88)
Lithuania	0.6 (0.4 to 0.8)	2.3 (1.2 to 3.8)	113.77 (7.17 to 326.38)	0 (0 to 0)
Luxembourg	0.7 (0.4 to 1)	1.2 (0.8 to 1.7)	49.94 (21.58 to 84.92)	0.02 (0.02 to 0.02)
Madagascar	1 (0.6 to 1.5)	2.6 (1.6 to 4)	140.63 (123.17 to 159.46)	-0.02 (-0.02 to -0.01)
Malawi	0.6 (0.4 to 0.9)	2.7 (1.6 to 4)	145.58 (28.07 to 370.91)	-0.02 (-0.02 to -0.02)
Malaysia	0.5 (0.3 to 0.8)	1 (0.6 to 1.5)	40.69 (-1.88 to 101.73)	0.01 (0.01 to 0.01)
Maldives	0.7 (0.4 to 1)	1.3 (0.8 to 2.3)	58.95 (18.91 to 112.46)	0.01 (0.01 to 0.02)
Mali	1.2 (0.7 to 1.8)	2.5 (1.4 to 3.8)	114.72	0.01

Supplementary Table 2 Years lived with disability of Guillain–Barré syndrome in 2020 and 2021 and the estimated annual percentage change in the age-standardized rates per 100,000 in 204 countries and territories, from 1990 to 2021

Country and territory	ASR in 2020 (per 100,000)	ASR in 2021 (per 100,000)	2019-2021 EAPC in ASR	1990-2019 EAPC in ASR
			(111.14 to 118.35)	(0.01 to 0.01)
Malta	0.6 (0.4 to 0.9)	1 (0.7 to 1.4)	37.97<br>(5.31 to 80.77)	0.02<br>(0.02 to 0.03)
Marshall Islands	0.4 (0.2 to 0.6)	0.4 (0.2 to 0.6)	4.07<br>(0.14 to 8.16)	0.01<br>(0.01 to 0.02)
Mauritania	1 (0.6 to 1.5)	2.2 (1.2 to 3.8)	101.81<br>(71.15 to 137.96)	0.01<br>(0.01 to 0.01)
Mauritius	0.5 (0.3 to 0.8)	0.6 (0.4 to 0.9)	10.66<br>(1.42 to 20.75)	0<br>(0 to 0)
Mexico	1.9 (1.2 to 2.7)	3.1 (2 to 4.5)	48.86<br>(31.46 to 68.56)	0.06<br>(0.05 to 0.08)
Micronesia (Federated States of)	0.4 (0.2 to 0.6)	0.4 (0.2 to 0.6)	0.03<br>(0 to 0.07)	0<br>(-0.01 to 0.01)
Monaco	0.6 (0.4 to 0.9)	1 (0.7 to 1.5)	40.04<br>(4.98 to 86.82)	0.01<br>(0.01 to 0.01)
Mongolia	0.5 (0.4 to 0.8)	1.4 (0.9 to 2.1)	60.72<br>(-5.03 to 172)	0<br>(0 to 0)
Montenegro	0.6 (0.4 to 0.9)	3.1 (2 to 4.6)	188.69<br>(49.8 to 456.35)	-0.23<br>(-0.36 to -0.1)
Morocco	0.8 (0.5 to 1.2)	2.3 (1.1 to 3.9)	121.79<br>(63.73 to 200.43)	0<br>(0 to 0)
Mozambique	0.6 (0.4 to 0.9)	3 (1.8 to 4.6)	159.91<br>(21.56 to 455.72)	-0.01<br>(-0.02 to -0.01)
Myanmar	0.6 (0.4 to 0.9)	1.2 (0.7 to 1.8)	48.31<br>(5.17 to 109.14)	0<br>(0 to 0)
Namibia	0.6 (0.4 to 1)	2.1 (1.2 to 3.4)	99.03<br>(12.17 to 253.14)	-0.01<br>(-0.01 to -0.01)
Nauru	0.4 (0.3 to 0.6)	0.6 (0.4 to 1)	31.57<br>(10.97 to 55.99)	-0.03<br>(-0.03 to -0.03)
Nepal	0.8 (0.5 to 1.2)	2.5 (1.5 to 4)	108.97<br>(29.57 to 237.05)	-0.54<br>(-0.68 to -0.39)
Netherlands	0.7 (0.4 to 1)	1.2 (0.7 to 1.8)	50.65<br>(27.96 to 77.36)	0.81<br>(0.57 to 1.05)
New Zealand	0.6 (0.4 to 0.9)	0.6 (0.4 to 0.9)	0.24<br>(0.23 to 0.26)	-0.19<br>(-0.26 to -0.12)
Nicaragua	1.5 (1 to 2.1)	2.5 (1.6 to 3.7)	48<br>(24.62 to 75.76)	0.07<br>(-0.01 to 0.14)
Niger	1 (0.6 to 1.5)	1.9 (1.1 to 3)	85.5<br>(77.94 to 93.39)	-0.01<br>(-0.01 to -0.01)
Nigeria	1.3 (0.9 to 2)	2.5 (1.6 to 3.7)	104.17<br>(86.49 to 123.52)	-0.02<br>(-0.03 to -0.01)
Niue	0.4 (0.2 to 0.6)	0.4 (0.2 to 0.6)	-0.01<br>(-0.01 to -0.01)	0.01<br>(0 to 0.01)
North Macedonia	0.8 (0.5 to 1.2)	3.4 (2 to 5.1)	182.69<br>(86.85 to 327.68)	0<br>(0 to 0)
Northern Mariana Islands	0.4 (0.3 to 0.7)	0.6 (0.4 to 0.8)	22.38<br>(17.34 to 27.64)	-0.01<br>(-0.01 to 0)
Norway	1.1 (0.7 to 1.5)	1.2 (0.8 to 1.8)	10.33<br>(4.3 to 16.72)	0.26<br>(0.13 to 0.4)
Oman	0.8 (0.5 to 1.1)	1.5 (0.9 to 2.4)	80.41<br>(59.13 to 104.55)	0<br>(0 to 0)
Pakistan	1.3 (0.8 to 1.9)	2.4 (1.5 to 3.7)	82.22<br>(74.98 to 89.76)	0.01<br>(0.01 to 0.01)
Palau	0.4 (0.2 to 0.6)	0.4 (0.2 to 0.6)	0.2<br>(0 to 0.41)	0.01<br>(0.01 to 0.02)
Palestine	0.7 (0.5 to 1.1)	3 (1.6 to 4.8)	153.17<br>(40.94 to 354.77)	0<br>(0 to 0)
Panama	1.5 (0.9 to 2.2)	2.3 (1.5 to 3.3)	40<br>(26.82 to 54.54)	0.16<br>(-0.01 to 0.34)
Papua New Guinea	0.4 (0.2 to 0.6)	1 (0.6 to 1.7)	66.79<br>(-2.9 to 186.49)	0<br>(0 to 0)
Paraguay	0.5 (0.3 to 0.8)	2.1 (1.2 to 3.3)	134.86<br>(24.47 to 343.17)	0.02<br>(-0.01 to 0.05)
Peru	1.4 (0.9 to 2.1)	2.5 (1.5 to 3.8)	75.7<br>(75.54 to 75.85)	0.06<br>(0.01 to 0.1)
Philippines	0.8 (0.5 to 1.2)	1.7 (1.1 to 2.5)	58.79<br>(20.96 to 108.47)	0.48<br>(0.35 to 0.61)
Poland	0.6 (0.4 to 0.8)	1.9 (1.2 to 2.9)	112.45<br>(21.24 to 272.28)	-0.35<br>(-0.42 to -0.27)
Portugal	0.6 (0.4 to 0.9)	1.2 (0.8 to 1.7)	52.3<br>(12.39 to 106.39)	0<br>(0 to 0.01)
Puerto Rico	0.5 (0.3 to 0.8)	0.8 (0.5 to 1.2)	32.2<br>(8.55 to 60.99)	-0.74<br>(-1.21 to -0.27)

Supplementary Table 2 Years lived with disability of Guillain–Barré syndrome in 2020 and 2021 and the estimated annual percentage change in the age-standardized rates per 100,000 in 204 countries and territories, from 1990 to 2021

Country and territory	ASR in 2020 (per 100,000)	ASR in 2021 (per 100,000)	2019-2021 EAPC in ASR	1990-2019 EAPC in ASR
Qatar	1.2 (0.7 to 1.9)	2 (1.1 to 3)	102.7 (54.37 to 166.16)	0.01 (0.01 to 0.01)
Republic of Korea	1.7 (1.1 to 2.6)	1.8 (1.1 to 2.7)	1.06 (0.18 to 1.94)	0.01 (0 to 0.01)
Republic of Moldova	0.9 (0.5 to 1.3)	2.6 (1.4 to 4.3)	128.37 (75.14 to 197.76)	0 (0 to 0.01)
Romania	0.6 (0.4 to 0.9)	2.1 (1.1 to 3.2)	121.03 (43.69 to 239.98)	0 (0 to 0)
Russian Federation	0.9 (0.6 to 1.3)	2.6 (1.6 to 3.8)	118.77 (60.47 to 198.25)	0.01 (0.01 to 0.01)
Rwanda	0.5 (0.3 to 0.7)	1.4 (0.9 to 2.3)	79.9 (1.34 to 219.38)	-0.01 (-0.02 to 0)
Saint Kitts and Nevis	0.6 (0.4 to 1)	0.8 (0.5 to 1.1)	11.25 (1.1 to 22.43)	0.35 (-0.06 to 0.76)
Saint Lucia	0.6 (0.4 to 0.9)	1.1 (0.6 to 1.7)	33.64 (-2.25 to 82.72)	0.14 (-0.02 to 0.29)
Saint Vincent and the Grenadines	0.6 (0.4 to 1)	0.9 (0.6 to 1.4)	20.78 (0.13 to 45.68)	0.16 (-0.02 to 0.34)
Samoa	0.4 (0.2 to 0.6)	0.4 (0.2 to 0.6)	0.87 (0.01 to 1.74)	0 (0 to 0)
San Marino	0.9 (0.6 to 1.3)	1.7 (1.1 to 2.4)	79.58 (70.85 to 88.74)	-0.01 (-0.01 to -0.01)
Sao Tome and Principe	1.2 (0.7 to 1.9)	1.5 (0.9 to 2.3)	67.53 (25.21 to 124.16)	0.01 (0.01 to 0.01)
Saudi Arabia	0.8 (0.5 to 1.2)	1 (0.5 to 1.5)	41.9 (19.36 to 68.7)	0 (0 to 0.01)
Senegal	0.9 (0.6 to 1.4)	2.8 (1.7 to 4.2)	125.4 (64 to 209.78)	0 (0 to 0)
Serbia	0.4 (0.3 to 0.6)	1.9 (1.2 to 3)	171.86 (43.63 to 414.56)	-0.49 (-0.77 to -0.21)
Seychelles	0.5 (0.3 to 0.8)	1.7 (1.1 to 2.5)	78.18 (-4.26 to 231.61)	0.01 (0.01 to 0.01)
Sierra Leone	1.2 (0.7 to 1.9)	1.3 (0.8 to 2)	55.21 (4.89 to 129.68)	0 (0 to 0.01)
Singapore	1.8 (1.1 to 2.7)	1.8 (1.2 to 2.7)	2.16 (1.23 to 3.09)	0.02 (0.01 to 0.03)
Slovakia	0.5 (0.3 to 0.7)	1.8 (1.1 to 2.8)	108.68 (4.86 to 315.31)	0.01 (0 to 0.01)
Slovenia	0.5 (0.3 to 0.8)	1.6 (0.9 to 2.9)	94.97 (11.9 to 239.72)	0.03 (0.02 to 0.03)
Solomon Islands	0.4 (0.2 to 0.6)	0.4 (0.2 to 0.6)	1.59 (0.29 to 2.91)	-0.01 (-0.01 to -0.01)
Somalia	0.8 (0.5 to 1.4)	2 (1.2 to 3.1)	113.36 (82.8 to 149.04)	-0.02 (-0.02 to -0.02)
South Africa	0.9 (0.6 to 1.3)	2.3 (1.4 to 3.3)	93.8 (47.19 to 155.16)	0.01 (0 to 0.01)
South Sudan	0.9 (0.5 to 1.6)	1.7 (0.9 to 2.9)	91.75 (78.54 to 105.94)	-0.02 (-0.03 to -0.02)
Spain	0.7 (0.5 to 1)	1.3 (0.9 to 1.9)	58.77 (36.74 to 84.34)	2.11 (1.74 to 2.48)
Sri Lanka	0.5 (0.3 to 0.8)	0.8 (0.5 to 1.2)	23.31 (-1.15 to 53.83)	-0.01 (-0.01 to -0.01)
Sudan	1.1 (0.6 to 1.8)	1.7 (0.8 to 2.8)	89.26 (48.01 to 141.99)	0 (0 to 0)
Suriname	0.7 (0.5 to 1.1)	1.6 (0.9 to 2.5)	59.13 (15.08 to 120.04)	0.15 (-0.03 to 0.33)
Sweden	1 (0.6 to 1.4)	1.4 (0.9 to 2)	33.31 (21.45 to 46.32)	2.2 (1.88 to 2.53)
Switzerland	0.6 (0.4 to 0.9)	1.1 (0.7 to 1.5)	41.74 (14.35 to 75.69)	0.02 (0.01 to 0.02)
Syrian Arab Republic	0.5 (0.3 to 0.8)	0.8 (0.4 to 1.3)	26.37 (3.18 to 54.78)	-0.01 (-0.01 to 0)
Taiwan (Province of China)	0.3 (0.2 to 0.4)	0.3 (0.2 to 0.4)	2.36 (0.88 to 3.86)	0.68 (0.58 to 0.79)
Tajikistan	0.9 (0.6 to 1.3)	2.2 (1.3 to 3.4)	99.9 (53.63 to 160.12)	0.03 (0.03 to 0.03)
Thailand	0.5 (0.3 to 0.8)	0.7 (0.4 to 1.1)	14.6 (-1.22 to 32.95)	0 (0 to 0)
Timor-Leste	0.5 (0.3 to 0.8)	1 (0.6 to 1.6)	37.94 (-3.72 to 97.62)	0 (0 to 0)
Togo	0.7 (0.5 to 1.1)	1.9 (1.1 to 2.9)	85.57 (29.39 to 166.16)	-0.02 (-0.02 to -0.02)
Tokelau	0.4 (0.2 to 0.6)	0.4 (0.2 to 0.6)	-0.01	0.01

Supplementary Table 2 Years lived with disability of Guillain–Barré syndrome in 2020 and 2021 and the estimated annual percentage change in the age-standardized rates per 100,000 in 204 countries and territories, from 1990 to 2021

Country and territory	ASR in 2020 (per 100,000)	ASR in 2021 (per 100,000)	2019-2021 EAPC in ASR	1990-2019 EAPC in ASR
			(-0.01 to 0)	(0 to 0.01)
Tonga	0.4 (0.2 to 0.6)	0.4 (0.2 to 0.6)	0.14 (-0.02 to 0.3)	0 (0 to 0)
Trinidad and Tobago	0.7 (0.4 to 1)	1 (0.7 to 1.5)	28.45 (3.17 to 59.92)	0.03 (0 to 0.06)
Tunisia	0.6 (0.4 to 0.9)	2.8 (1.5 to 4.5)	141.13 (18.82 to 389.34)	0 (0 to 0)
Turkey	0.9 (0.5 to 1.4)	1.7 (1 to 2.8)	76.21 (55.02 to 100.31)	0.06 (0.05 to 0.08)
Turkmenistan	0.9 (0.5 to 1.3)	2.2 (1.3 to 3.6)	101.69 (54.34 to 163.57)	0.02 (0.01 to 0.02)
Tuvalu	0.4 (0.3 to 0.6)	0.7 (0.4 to 1)	32.2 (11.32 to 57)	0.02 (0.02 to 0.02)
Uganda	0.5 (0.3 to 0.8)	1.7 (1.1 to 2.6)	97.54 (11.95 to 248.56)	-0.02 (-0.02 to -0.01)
Ukraine	0.7 (0.5 to 1.1)	2.2 (1.2 to 3.8)	100.73 (23.31 to 226.76)	0.01 (0.01 to 0.01)
United Arab Emirates	0.7 (0.5 to 1.1)	1.7 (1 to 2.8)	90.51 (49.12 to 143.39)	0.02 (0.02 to 0.02)
United Kingdom	0.6 (0.4 to 0.9)	1.1 (0.7 to 1.6)	53.7 (37.96 to 71.23)	2.65 (2.23 to 3.07)
United Republic of Tanzania	0.7 (0.5 to 1)	2.2 (1.4 to 3.2)	127.34 (57.44 to 228.29)	0.78 (0.63 to 0.94)
United States of America	1.4 (0.9 to 2)	2.1 (1.4 to 3.1)	30.22 (11.24 to 52.44)	0.44 (0.32 to 0.56)
United States Virgin Islands	0.7 (0.5 to 1.1)	1.1 (0.7 to 1.7)	34.31 (16.34 to 55.06)	0.14 (-0.04 to 0.31)
Uruguay	1 (0.6 to 1.5)	1.6 (1 to 2.3)	27.12 (-2.16 to 65.17)	0 (0 to 0)
Uzbekistan	1 (0.6 to 1.5)	1.8 (0.9 to 2.9)	83.24 (81.83 to 84.66)	0.02 (0.02 to 0.02)
Vanuatu	0.4 (0.2 to 0.6)	0.4 (0.3 to 0.6)	3.17 (-0.42 to 6.87)	-0.01 (-0.01 to -0.01)
Venezuela (Bolivarian Republic of)	1.4 (0.9 to 2.1)	2.3 (1.5 to 3.5)	42.7 (21.39 to 67.74)	0.06 (-0.02 to 0.14)
Viet Nam	0.5 (0.3 to 0.8)	0.6 (0.4 to 0.9)	9.26 (-0.99 to 20.57)	0.01 (0 to 0.01)
Yemen	0.9 (0.5 to 1.4)	1.3 (0.6 to 2.3)	63.47 (39.22 to 91.94)	0 (0 to 0)
Zambia	0.7 (0.5 to 1.1)	2.7 (1.6 to 4)	144.76 (58.15 to 278.8)	-0.03 (-0.03 to -0.02)
Zimbabwe	0.7 (0.4 to 1)	2.6 (1.4 to 4.1)	121.2 (20.54 to 305.93)	-0.01 (-0.02 to -0.01)

Notes: Countries and territories were ordered alphabetically. ASR=age-standardized rate. CI=confidence interval. EAPC=estimated annual percentage change. GBD=Global Burden of Diseases, Injuries, and Risk Factors Study. SDI=Socio-demographic index. UI=uncertainty interval. YLD=years lived with disability.

Supplementary Table 3 Male and female age-standardized years lived with disability rates (ASYRs) attributed to COVID-19, and ASYR sex ratio, in 2020 and 2021

		2020			2021		
**GBD super regions**	**GBD regions**	**ASYR in males (per 100,000)**	**ASYR in females (per 100,000)**	**ASYR male to female ratio**	**ASYR in males (per 100,000)**	**ASYR in females (per 100,000)**	**ASYR male to female ratio**
Global		0.25 (0.16 to 0.39)	0.25 (0.16 to 0.38)	1.00	1.15 (0.72 to 1.73)	1.14 (0.71 to 1.71)	1.01
Low SDI		0.44 (0.27 to 0.66)	0.42 (0.26 to 0.63)	1.05	1.83 (1.12 to 2.77)	1.77 (1.07 to 2.66)	1.03
Low-middle SDI		0.4 (0.25 to 0.6)	0.39 (0.24 to 0.58)	1.03	1.7 (1.02 to 2.49)	1.64 (0.98 to 2.41)	1.04
Middle SDI		0.21 (0.13 to 0.31)	0.2 (0.13 to 0.31)	1.05	0.97 (0.6 to 1.43)	0.95 (0.58 to 1.41)	1.02
High-middle SDI		0.12 (0.07 to 0.19)	0.13 (0.07 to 0.19)	0.92	0.71 (0.44 to 1.08)	0.73 (0.46 to 1.11)	0.97
High SDI		0.12 (0.07 to 0.17)	0.11 (0.07 to 0.16)	1.09	0.58 (0.36 to 0.85)	0.56 (0.35 to 0.83)	1.04
Central Europe, eastern Europe, and central Asia		0.29 (0.18 to 0.43)	0.28 (0.18 to 0.42)	1.04	1.82 (1.13 to 2.72)	1.75 (1.1 to 2.65)	1.04
	Central Asia	0.36 (0.2 to 0.57)	0.35 (0.19 to 0.56)	1.03	1.55 (0.91 to 2.36)	1.49 (0.86 to 2.29)	1.04
	Central Europe	0.16 (0.1 to 0.25)	0.16 (0.09 to 0.24)	1.00	1.77 (1.07 to 2.62)	1.7 (1.03 to 2.51)	1.04
	Eastern Europe	0.32 (0.2 to 0.48)	0.31 (0.19 to 0.46)	1.03	1.99 (1.23 to 3.05)	1.91 (1.19 to 2.95)	1.04
Hign-income		0.12 (0.07 to 0.17)	0.11 (0.07 to 0.17)	1.09	0.57 (0.36 to 0.84)	0.56 (0.35 to 0.82)	1.02
	Australasia	0 (0 to 0.01)	0 (0 to 0.01)	NA	0.01 (0.01 to 0.02)	0.01 (0.01 to 0.02)	1.00
	High-income Asia Pacific	0.01 (0.01 to 0.02)	0.01 (0.01 to 0.02)	1.00	0.1 (0.06 to 0.15)	0.1 (0.06 to 0.15)	1.00
	High-income North America	0.15 (0.09 to 0.23)	0.15 (0.09 to 0.22)	1.00	0.83 (0.52 to 1.23)	0.8 (0.49 to 1.18)	1.04
	Southern Latin America	0.12 (0.07 to 0.18)	0.11 (0.07 to 0.18)	1.09	0.66 (0.38 to 1.04)	0.64 (0.37 to 1.01)	1.03
	Western Europe	0.13 (0.08 to 0.2)	0.13 (0.08 to 0.19)	1.00	0.56 (0.35 to 0.84)	0.54 (0.34 to 0.81)	1.04
Latin America and Caribbean		0.41 (0.25 to 0.6)	0.39 (0.24 to 0.59)	1.05	1.55 (0.94 to 2.31)	1.5 (0.91 to 2.25)	1.03
	Andean Latin America	0.66 (0.39 to 1.02)	0.64 (0.38 to 1)	1.03	1.91 (1.14 to 2.95)	1.86 (1.13 to 2.87)	1.03
	Caribbean	0.15 (0.07 to 0.25)	0.15 (0.07 to 0.24)	1.00	0.54 (0.3 to 0.85)	0.53 (0.29 to 0.83)	1.02
	Tropical Latin America	0.41 (0.26 to 0.61)	0.4 (0.25 to 0.59)	1.02	1.64 (1.01 to 2.45)	1.57 (0.98 to 2.38)	1.04
	Central Latin America	0.39 (0.23 to 0.57)	0.37 (0.23 to 0.56)	1.05	1.56 (0.95 to 2.32)	1.51 (0.92 to 2.26)	1.03
North Africa and Middle East	North Africa and Middle East	0.44 (0.25 to 0.68)	0.42 (0.24 to 0.67)	1.05	1.44 (0.87 to 2.19)	1.41 (0.84 to 2.12)	1.02
South Asia	South Asia	0.38 (0.23 to 0.58)	0.37 (0.22 to 0.57)	1.03	1.82 (1.11 to 2.74)	1.75 (1.07 to 2.63)	1.04
Southeast Asia, east Asia, and Oceania		0.03 (0.02 to 0.05)	0.03 (0.02 to 0.05)	1.00	0.29 (0.18 to 0.43)	0.29 (0.18 to 0.43)	1.00
	Southeast Asia	0.09 (0.05 to 0.13)	0.08 (0.05 to 0.12)	1.12	0.84 (0.52 to 1.24)	0.8 (0.5 to 1.19)	1.05
	East Asia	0.01 (0 to 0.01)	0.01 (0 to 0.01)	1.00	0 (0 to 0)	0 (0 to 0)	NA
	Oceania	0.02 (0.01 to 0.03)	0.01 (0.01 to 0.03)	1.00	0.59 (0.28 to 1.05)	0.56 (0.27 to 1)	1.05
Sub-Saharan Africa		0.47 (0.3 to 0.71)	0.46 (0.29 to 0.69)	1.02	1.91 (1.19 to 2.85)	1.84 (1.15 to 2.75)	1.04
	Western Sub-Saharan Africa	0.61 (0.38 to 0.93)	0.59 (0.37 to 0.9)	1.03	1.77 (1.11 to 2.67)	1.7 (1.06 to 2.58)	1.04

Supplementary Table 3 Male and female age-standardized years lived with disability rates (ASYRs) attributed to COVID-19, and ASYR sex ratio, in 2020 and 2021

		2020			2021		
**GBD super regions**	**GBD regions**	**ASYR in males (per 100,000)**	**ASYR in females (per 100,000)**	**ASYR male to female ratio**	**ASYR in males (per 100,000)**	**ASYR in females (per 100,000)**	**ASYR male to female ratio**
	Eastern Sub-Saharan Africa	0.31 (0.19 to 0.46)	0.3 (0.19 to 0.44)	1.03	2.03 (1.26 to 3.01)	1.96 (1.21 to 2.92)	1.04
	Central Sub-Saharan Africa	0.63 (0.36 to 1)	0.61 (0.34 to 0.95)	1.03	2.14 (1.26 to 3.2)	2.07 (1.22 to 3.09)	1.03
	Southern Sub-Saharan Africa	0.27 (0.17 to 0.4)	0.26 (0.16 to 0.38)	1.04	1.76 (1.1 to 2.58)	1.71 (1.07 to 2.53)	1.03

NA=not applicable.

Supplementary Table 4 Dependent and independent variables in generalized linear models

Variables	Year	n (% of total)	Median (IQR)
Change in GBS burden of YLD rate (%)	2019-2021	204 (100.0)	172.7 (74.34 to 321.65)
Total vaccinations (per_hundred)	by 31.12.2021	183 (89.7)	101.14 (40.12 to 155.88)
People vaccinated (per hundred)	by 31.12.2021	181 (88.7)	52.15 (25.61 to 72.33)
People fully vaccinated (per hundred)	by 31.12.2021	179 (87.8)	43.51 (18.54 to 67.5)
Median age (years)	2021	190 (93.1)	29.02 (21.02 to 38.3)
Female proportion (%)	2021	189 (92.7)	50.36 (49.7 to 50.98)
Governance stringency index	2021	175 (85.8)	57.74 (48.87 to 65.75)
COVID-19 incidence (per 100,000)	2021	204 (100.0)	31044.47 (15068.63 to 45866.67)
Social-demographic index	2021	203 (99.5)	0.67 (0.52 to 0.79)
No. of healthcare worker (per 10,000)	2019	203 (99.5)	114.69 (51.67 to 229.86)
Share of urban population (%)	2021	178 (87.3)	63.67 (42.61 to 77.62)
Per square kilometer population	2021	190 (93.1)	84.69 (32.08 to 204.71)
Per capita total expenditure on health (PPP int. $)	2021	201 (98.5)	1061 (294 to 2632)

Supplementary Table 5 Cause-specific age-standardized years lived with disability of Guillain-Barré syndrome at global and supper-regional levels, before and during the years of COVID-19 pandemic

Locations	Causes/triggers	2019	2020	2021
Global	COVID-19	0 (0 to 0)	0.25 (0.16 to 0.38)	1.14 (0.71 to 1.72)
	Diarrheal diseases	0.07 (0.04 to 0.11)	0.07 (0.04 to 0.11)	0.07 (0.04 to 0.11)
	Lower respiratory infections	0.07 (0.04 to 0.12)	0.07 (0.04 to 0.12)	0.07 (0.04 to 0.12)
	Other neurological disorders	0.23 (0.13 to 0.35)	0.23 (0.13 to 0.36)	0.23 (0.13 to 0.36)
	Other unspecified infectious diseases	0.04 (0.02 to 0.07)	0.04 (0.02 to 0.07)	0.04 (0.02 to 0.07)
	Upper respiratory infections	0.19 (0.12 to 0.3)	0.19 (0.12 to 0.3)	0.19 (0.12 to 0.3)
	Zika virus	0 (0 to 0)	0 (0 to 0)	0 (0 to 0)
Central Europe, Easte rn Europe, and Centr al Asia	COVID-19	0 (0 to 0)	0.28 (0.18 to 0.42)	1.78 (1.11 to 2.68)
	Diarrheal diseases	0.06 (0.03 to 0.09)	0.06 (0.03 to 0.09)	0.06 (0.03 to 0.09)
	Lower respiratory infections	0.06 (0.03 to 0.1)	0.06 (0.03 to 0.1)	0.06 (0.03 to 0.1)
	Other neurological disorders	0.19 (0.11 to 0.3)	0.19 (0.11 to 0.3)	0.19 (0.11 to 0.3)
	Other unspecified infectious diseases	0.04 (0.02 to 0.06)	0.04 (0.02 to 0.06)	0.04 (0.02 to 0.06)
	Upper respiratory infections	0.16 (0.1 to 0.26)	0.16 (0.1 to 0.26)	0.16 (0.1 to 0.26)
	Zika virus	0 (0 to 0)	0 (0 to 0)	0 (0 to 0)
High-income	COVID-19	0 (0 to 0)	0.11 (0.07 to 0.17)	0.57 (0.35 to 0.83)
	Diarrheal diseases	0.11 (0.06 to 0.18)	0.11 (0.06 to 0.18)	0.11 (0.07 to 0.18)
	Lower respiratory infections	0.11 (0.06 to 0.19)	0.12 (0.06 to 0.19)	0.12 (0.06 to 0.19)
	Other neurological disorders	0.38 (0.21 to 0.58)	0.38 (0.21 to 0.58)	0.38 (0.21 to 0.59)
	Other unspecified infectious diseases	0.07 (0.04 to 0.11)	0.07 (0.04 to 0.11)	0.07 (0.04 to 0.11)
	Upper respiratory infections	0.32 (0.2 to 0.48)	0.32 (0.2 to 0.49)	0.32 (0.2 to 0.49)
	Zika virus	0 (0 to 0)	0 (0 to 0)	0 (0 to 0)
Latin America and Caribbean	COVID-19	0 (0 to 0)	0.4 (0.24 to 0.59)	1.52 (0.92 to 2.28)
	Diarrheal diseases	0.09 (0.06 to 0.15)	0.09 (0.05 to 0.15)	0.09 (0.05 to 0.15)
	Lower respiratory infections	0.1 (0.05 to 0.16)	0.1 (0.05 to 0.16)	0.1 (0.05 to 0.16)
	Other neurological disorders	0.32 (0.18 to 0.48)	0.32 (0.18 to 0.48)	0.32 (0.18 to 0.48)
	Other unspecified infectious diseases	0.06 (0.03 to 0.09)	0.06 (0.03 to 0.09)	0.06 (0.03 to 0.09)
	Upper respiratory infections	0.27 (0.17 to 0.41)	0.27 (0.17 to 0.41)	0.27 (0.17 to 0.41)
	Zika virus	0 (0 to 0.01)	0 (0 to 0)	0 (0 to 0)
North Africa and Mid dle East	COVID-19	0 (0 to 0)	0.43 (0.25 to 0.67)	1.43 (0.86 to 2.16)
	Diarrheal diseases	0.05 (0.03 to 0.09)	0.05 (0.03 to 0.09)	0.05 (0.03 to 0.09)
	Lower respiratory infections	0.06 (0.03 to 0.09)	0.06 (0.03 to 0.09)	0.06 (0.03 to 0.09)
	Other neurological disorders	0.18 (0.1 to 0.29)	0.19 (0.1 to 0.29)	0.19 (0.1 to 0.29)
	Other unspecified infectious diseases	0.03 (0.02 to 0.06)	0.03 (0.02 to 0.06)	0.03 (0.02 to 0.06)
	Upper respiratory infections	0.16 (0.09 to 0.25)	0.16 (0.09 to 0.25)	0.16 (0.09 to 0.25)
	Zika virus	0 (0 to 0)	0 (0 to 0)	0 (0 to 0)
South Asia	COVID-19	0 (0 to 0)	0.38 (0.23 to 0.57)	1.78 (1.09 to 2.68)
	Diarrheal diseases	0.08 (0.05 to 0.13)	0.08 (0.05 to 0.13)	0.08 (0.05 to 0.13)
	Lower respiratory infections	0.08 (0.04 to 0.14)	0.08 (0.04 to 0.14)	0.08 (0.04 to 0.14)
	Other neurological disorders	0.28 (0.15 to 0.44)	0.28 (0.15 to 0.44)	0.28 (0.15 to 0.44)
	Other unspecified infectious diseases	0.05 (0.03 to 0.08)	0.05 (0.03 to 0.08)	0.05 (0.03 to 0.08)
	Upper respiratory infections	0.23 (0.14 to 0.37)	0.23 (0.14 to 0.37)	0.23 (0.14 to 0.37)
	Zika virus	0 (0 to 0)	0 (0 to 0)	0 (0 to 0)
Southeast Asia, East Asia, and Oceania	COVID-19	0 (0 to 0)	0.03 (0.02 to 0.05)	0.29 (0.18 to 0.43)
	Diarrheal diseases	0.03 (0.02 to 0.06)	0.03 (0.02 to 0.06)	0.03 (0.02 to 0.06)
	Lower respiratory infections	0.04 (0.02 to 0.06)	0.04 (0.02 to 0.06)	0.04 (0.02 to 0.06)
	Other neurological disorders	0.12 (0.06 to 0.18)	0.12 (0.06 to 0.18)	0.12 (0.06 to 0.18)

Supplementary Table 5 Cause-specific age-standardized years lived with disability of Guillain-Barré syndrome at global and supper-regional levels, before and during the years of COVID-19 pandemic

Locations	Causes/triggers	2019	2020	2021
	Other unspecified infectious diseases	0.02 (0.01 to 0.04)	0.02 (0.01 to 0.04)	0.02 (0.01 to 0.04)
	Upper respiratory infections	0.1 (0.06 to 0.16)	0.1 (0.06 to 0.16)	0.1 (0.06 to 0.16)
	Zika virus	0 (0 to 0)	0 (0 to 0)	0 (0 to 0)
Sub-Saharan Africa	COVID-19	0 (0 to 0)	0.46 (0.29 to 0.7)	1.87 (1.16 to 2.8)
	Diarrheal diseases	0.06 (0.03 to 0.1)	0.06 (0.03 to 0.1)	0.06 (0.03 to 0.1)
	Lower respiratory infections	0.06 (0.03 to 0.1)	0.06 (0.03 to 0.1)	0.06 (0.03 to 0.1)
	Other neurological disorders	0.2 (0.11 to 0.32)	0.2 (0.11 to 0.32)	0.2 (0.11 to 0.32)
	Other unspecified infectious diseases	0.04 (0.02 to 0.06)	0.04 (0.02 to 0.06)	0.04 (0.02 to 0.06)
	Upper respiratory infections	0.17 (0.1 to 0.28)	0.17 (0.1 to 0.28)	0.17 (0.1 to 0.28)
	Zika virus	0 (0 to 0)	0 (0 to 0)	0 (0 to 0)

Notes: Other neurological disorders: idiopathic Guillain-Barré syndrome, the cause is unknown.

Supplementary Table 6 Sensitivity analysis for the association between COVID-19 vaccination coverage and age-standardized YLD rate at national level in 2021, using generalized linear model with Gaussian distribution and log-link function.

			All-cause age-standardized YLD rate				COVID-19-specific age-standardized YLD rate			
**Independent variable**	**Model**	**n**	***β***	**SE**	**t-value**	***p***	***β***	**SE**	**t-value**	***p***
No. of total vaccinations (per hundred people)#	Comprehensive model*	148	-0.0033	0.0005	-5.81	<.001	-0.0040	0.0009	-4.56	<.001
	Minimal sufficient model†	161	-0.0028	0.0007	-4.24	<.001	-0.0052	0.0010	-5.15	<.001
	Direct effect model‡	161	-0.0014	0.0005	-2.6	0.01	-0.0033	0.0007	-4.42	<.001
No. of people vaccinated (per hundred people)#	Comprehensive model*	137	-0.0066	0.0014	-4.73	<.001	-0.0070	0.0022	-3.13	0.002
	Minimal sufficient model†	150	-0.0064	0.0016	-3.90	<.001	-0.0118	0.0024	-4.87	<.001
	Direct effect model‡	150	-0.0032	0.0014	-2.38	0.019	-0.0078	0.0018	-4.32	<.001
No. of people fully vaccinated (per hundred people)#	Comprehensive model*	143	-0.0081	0.0015	-5.60	<.001	-0.0090	0.0023	-3.91	<.001
	Minimal sufficient model†	156	-0.0070	0.0017	-4.20	<.001	-0.0128	0.0025	-5.08	<.001
	Direct effect model‡	156	-0.0039	0.0014	-2.84	0.005	-0.0087	0.0019	-4.67	<.001

β=regression coefficient; COVID-19=coronavirus disease 2019; SE=standard error; YLD=years lived with disability.
# Data on December 31, 2021 were obtained. Missing values were filled by data within a 11-day window (5 days before and after December 31, 2021).
* Model was adjusted for median of government stringency index (GSI) in 2021, socio-demographic index (SDI) in 2021, and age-standardized YLD rate in 2020.
† Model was adjusted for SDI in 2021.
‡ Model was adjusted for SDI in 2021 and COVID-19 incidence in 2021.

Supplementary Table 7 Sensitivity analysis of parameter estimates across different regression models and variable selection methods

Sensitivity analyses	Model	*β*	SE	*p*
Robust standard errors	DAG selected model	-0.27	0.05	<.0001
	LASSO selected model	-0.25	0.06	<.0001
	Full model	-0.22	0.06	<.0001
Gamma distribution with log link	DAG selected model	-0.30	0.07	<.0001
	LASSO selected model	-0.28	0.08	<.0001
	Full model	-0.26	0.08	< 0.001

Supplementary Table 8 Variance inflation factors for multicollinearity test of full model

Variables	VIF values
Median age	5.83
Female proportion	1.27
People vaccinated (per hundred)	3.44
Governance strigency index in 2021	1.47
COVID-19 incidence (per 100k) in 2021	1.54
Social-demographic index in 2021	6.46
Healthcare human resources	5.05
Rate of urbanization	2.06
Population density	1.07
Domestic health expenditure	4.07

2. Supplementary Figures

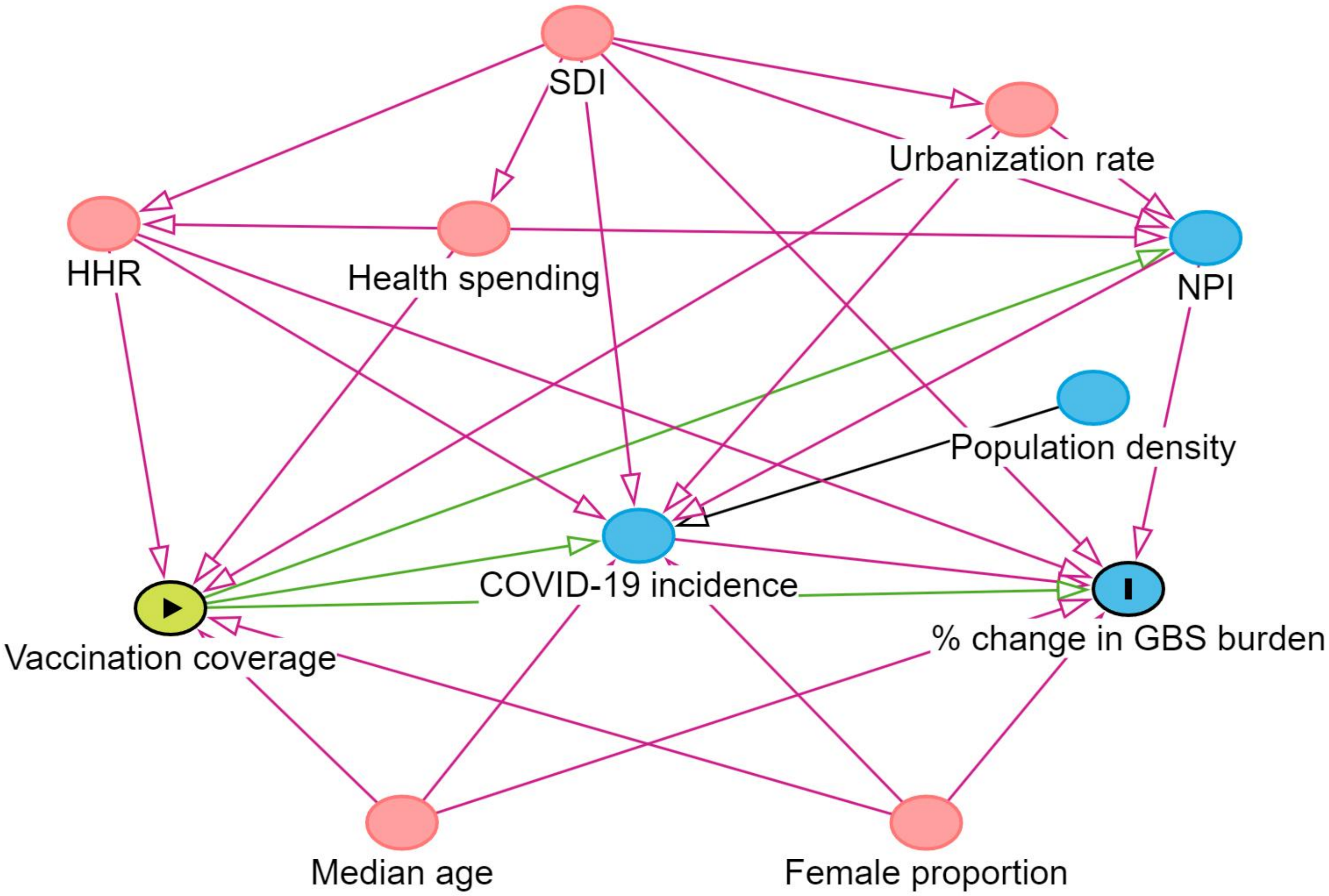

Supplementary Figure 1 Directed acyclic graph for the association between

GBS= Guillain-Barré syndrome; HHR=healthcare human resources, i.e. health worker density (per 10k); NPI=non-pharmacological interventions (government stringency index was used to represent the degree of NPI); SDI=socio-demographic index.

Notes: Pink line: biasing path. Green line: causal path. Pink pie: confounder (ancestor of exposure and outcome). Green pie: exposure/ancestor of exposure. Blue pie: ancestor of outcome.

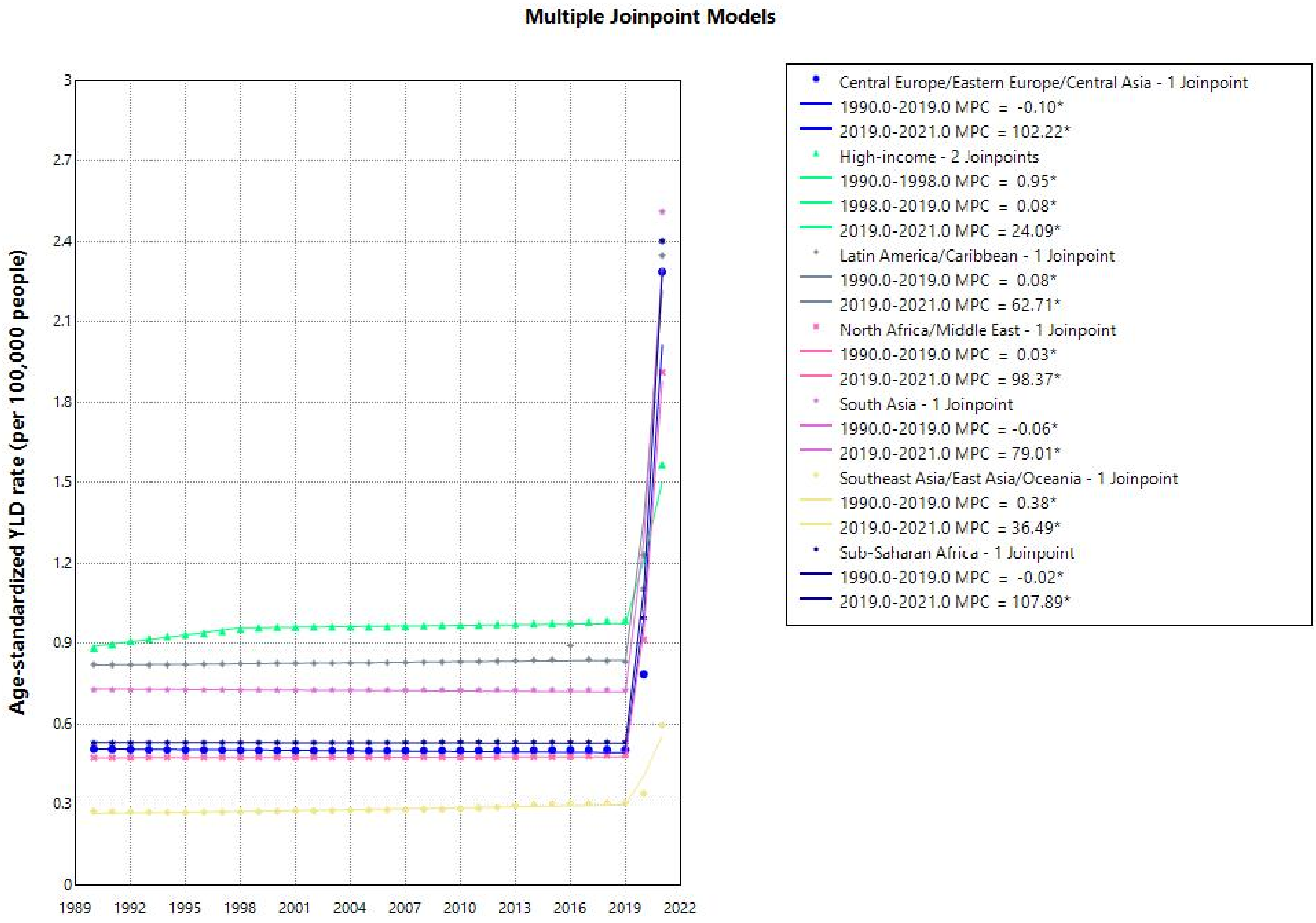

Supplementary Figure 2 Joinpoint regression analysis of the age-standardized years lived with disability rate of Guillain-Barré syndrome by seven GBD super regions, 1990–2021.

Notes: APC=annual percentage change; GBD=global burden of disease study; Asterisks represent those values that are statistically significantly different from 0.

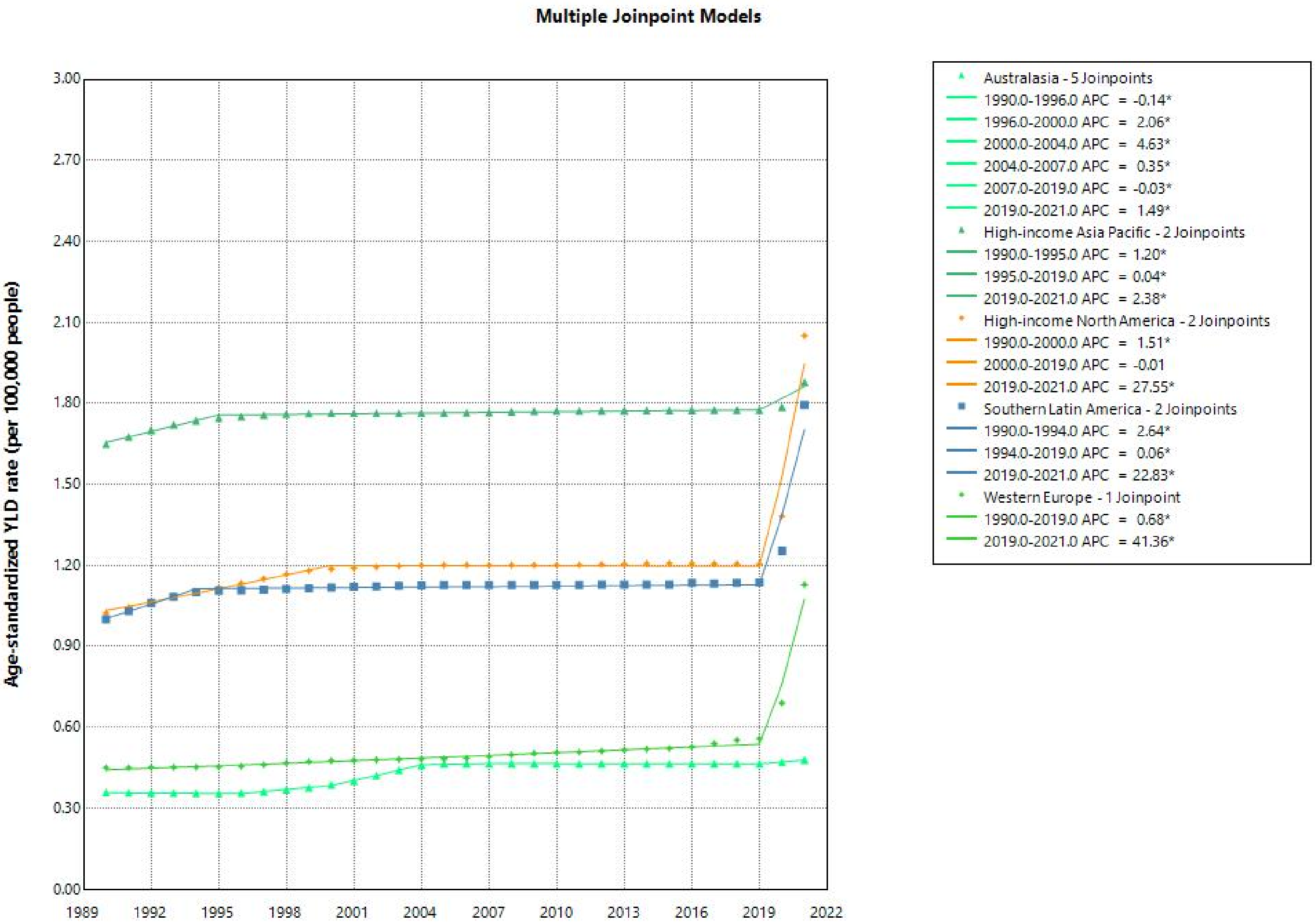

Supplementary Figure 3 Joinpoint regression analysis of the age-standardized years lived with disability rate of Guillain-Barré syndrome by High-income regions, 1990–2021.

Notes: APC=annual percentage change; Asterisks represent those values that are statistically significantly different from 0.

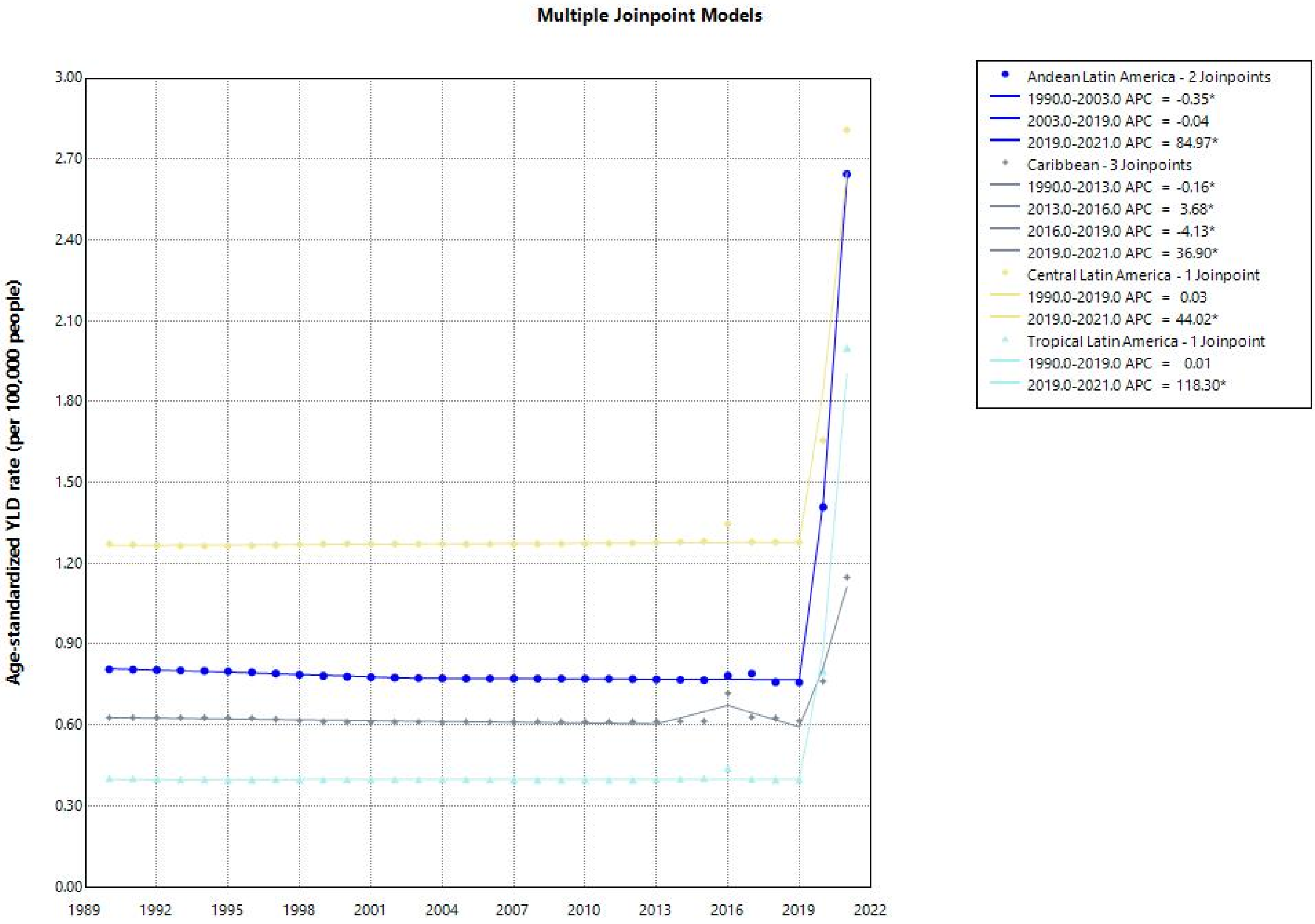

Supplementary Figure 4 Joinpoint regression analysis of the age-standardized years lived with disability rate of Guillain-Barré syndrome by Latin America regions, 1990–2021.
Notes: APC=annual percentage change; Asterisks represent those values that are statistically significantly different from 0.

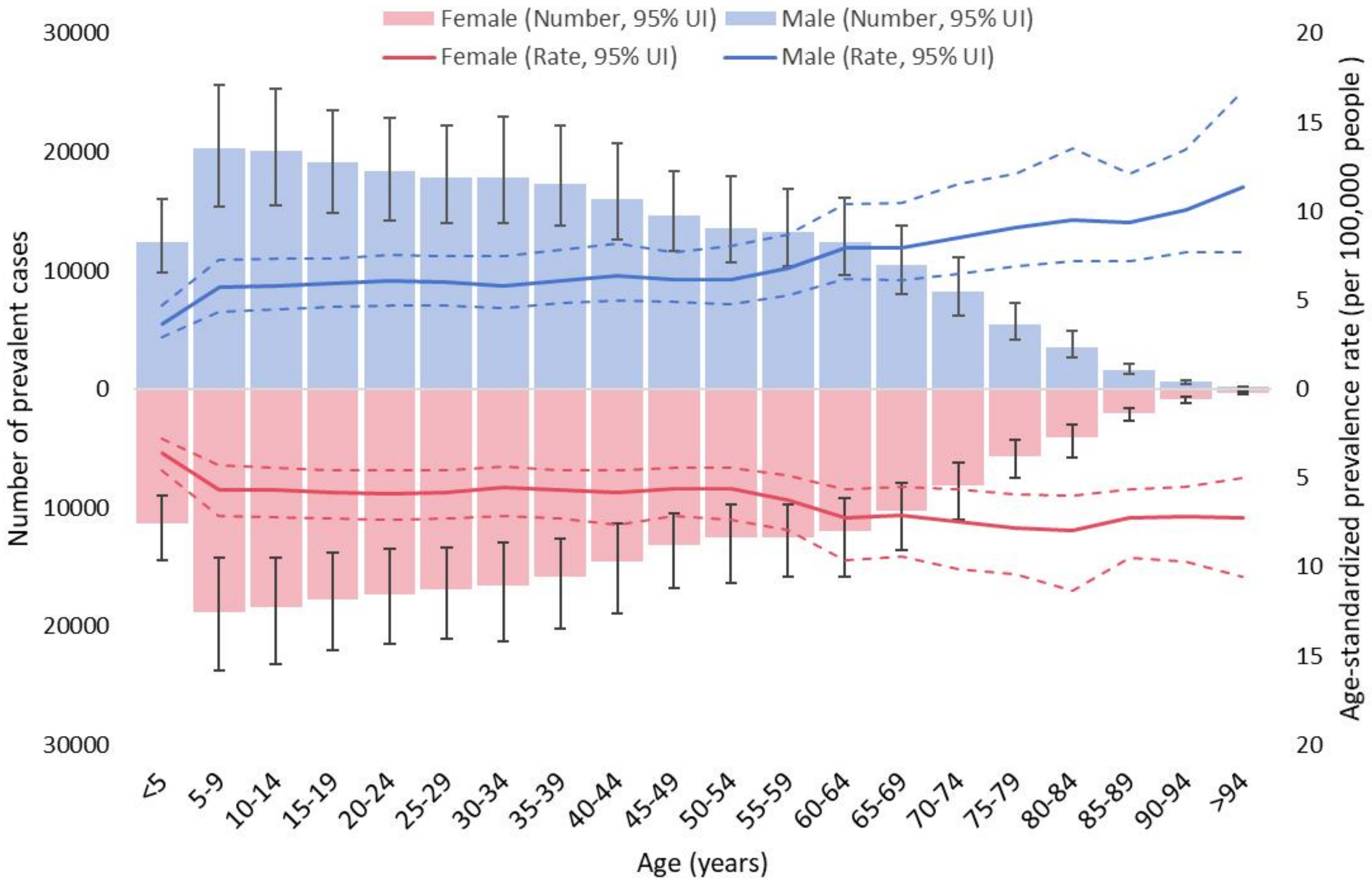

Supplementary Figure 5 Age-sex distribution of prevalence of Guillain-Barré syndrome in 2021

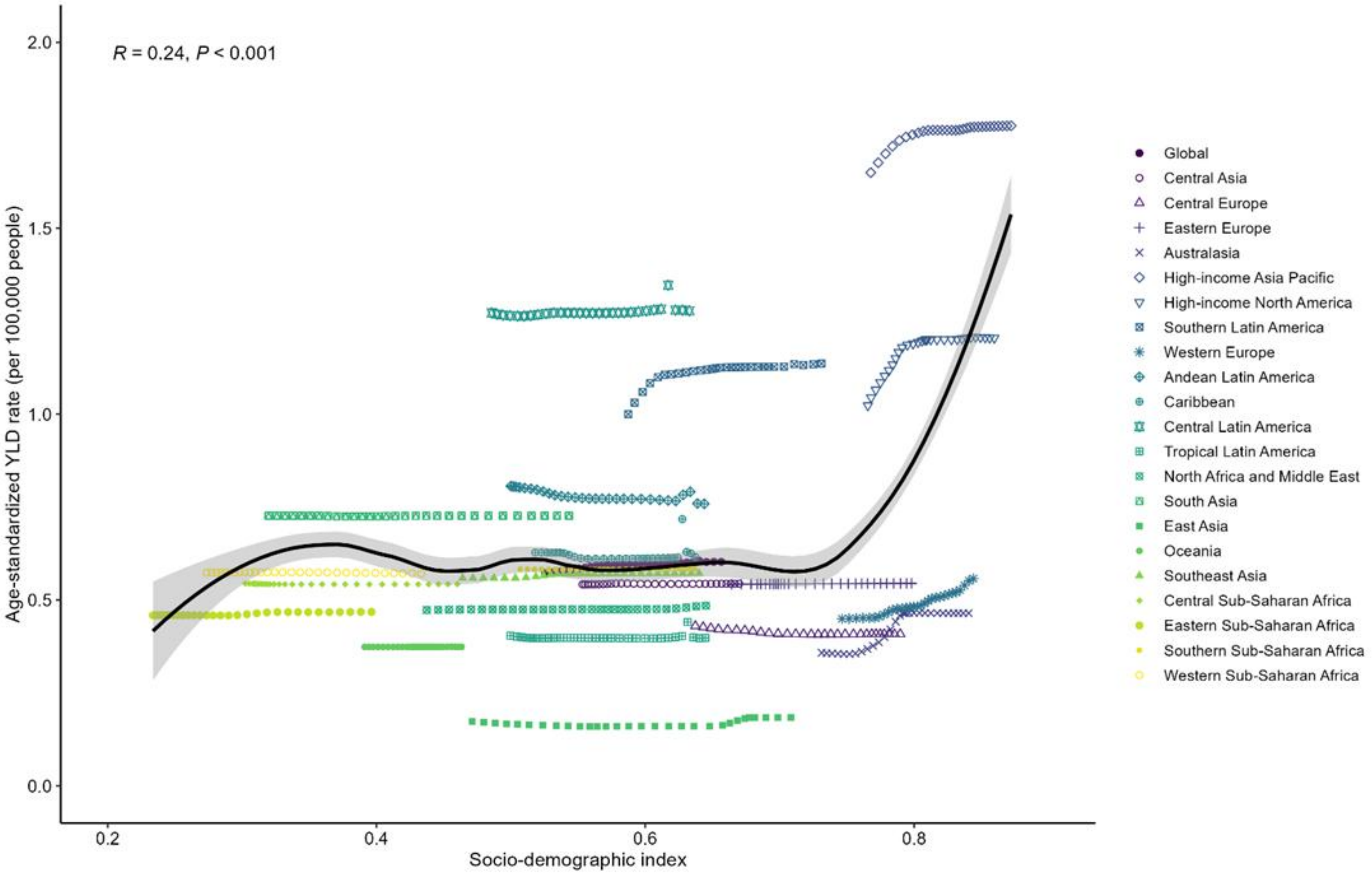

Supplementary Figure 6 Age-standardized years lived with disability rates (YLDs) of Guillain-Barré syndrome versus Socio-demographic Index, globally and across 21 regions. Each point shows the observed age-standardized YLDs rate for each region from 1990 to 2019.

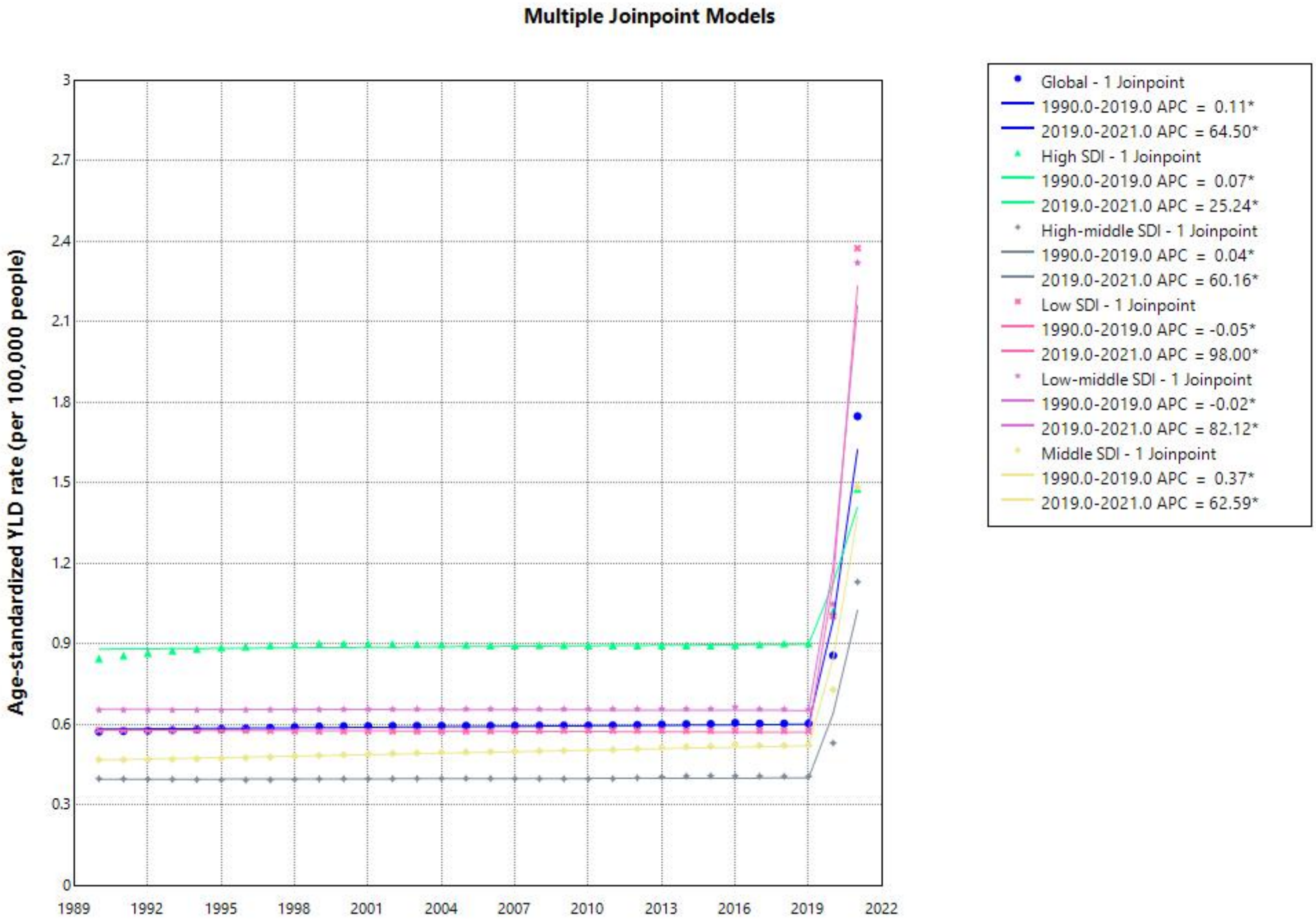

Supplementary Figure 7 Joinpoint regression analysis of the age-standardized years lived with disability rate of Guillain-Barré syndrome globally and by quintile of the socio-demographic index, 1990–2021.

Notes: APC=annual percentage change; Asterisks represent those values that are statistically significantly different from 0.

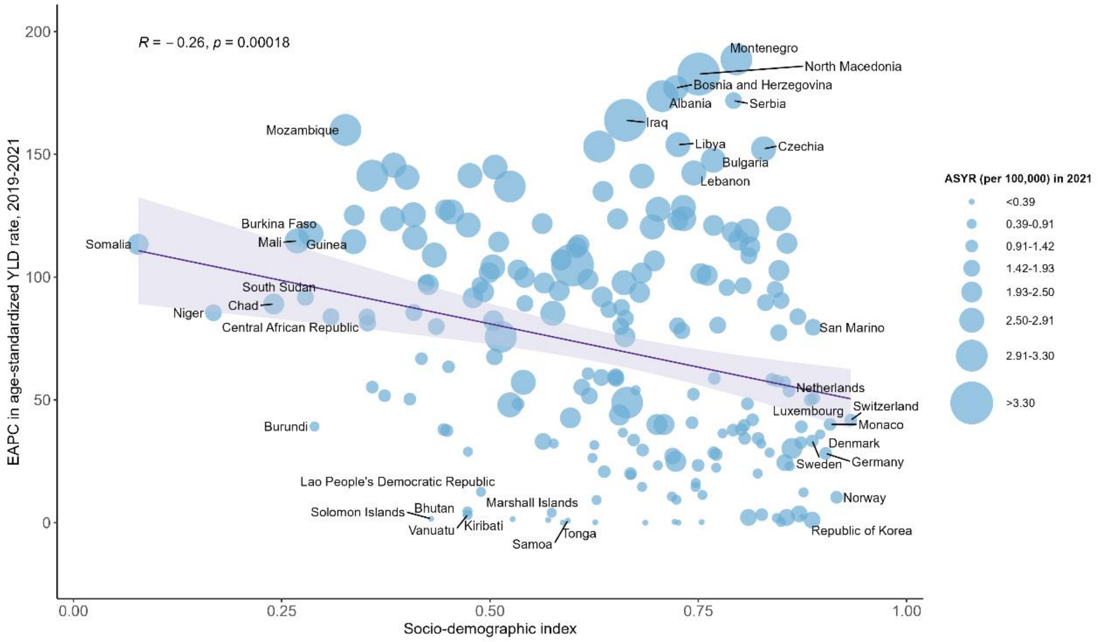

Supplementary Figure 8 Association between estimated annual percentage change in age-standardized years lived with disability rate during the COVID-19 pandemic, across 204 countries and territories.

ASYR=age-standardized years lived with disability rate; EAPC=estimated annual percentage change; YLD=years lived with disability.

Notes: Pearson correlation coefficient (R) and *p* value were presented to show the strength and direction of the association. Akaike Information Criterion (AIC) was used to compare the linear models and smooth models, and the AIC score indicated both models had similar performance.

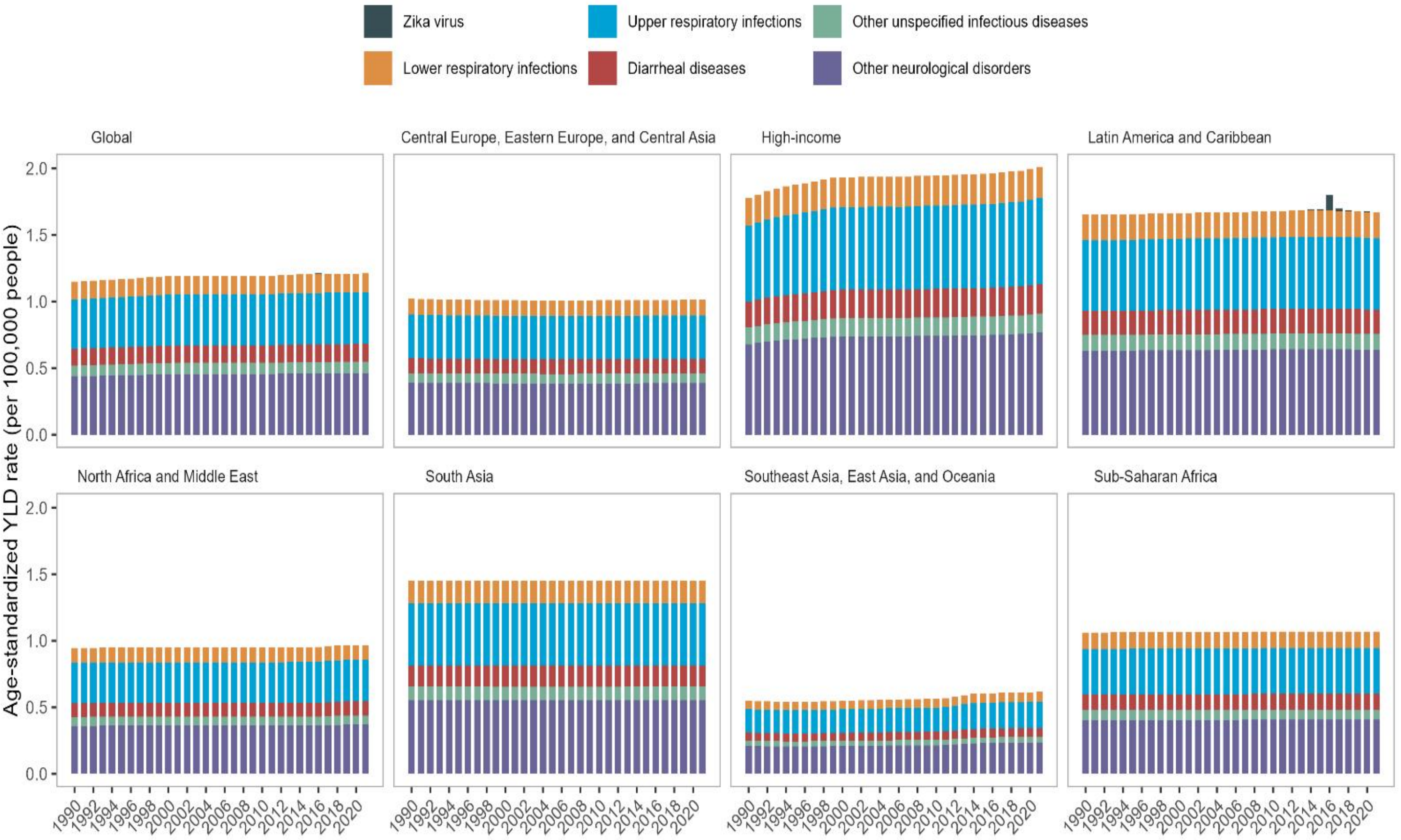

Supplementary Figure 9 Age-standardized years lived with disability rates of Guillain-Barré syndrome attributed to causes excluding COVID-19, by super region and years from 1990-2021.
Notes: Other neurological disorders: idiopathic Guillain-Barré syndrome, the cause is unknown.

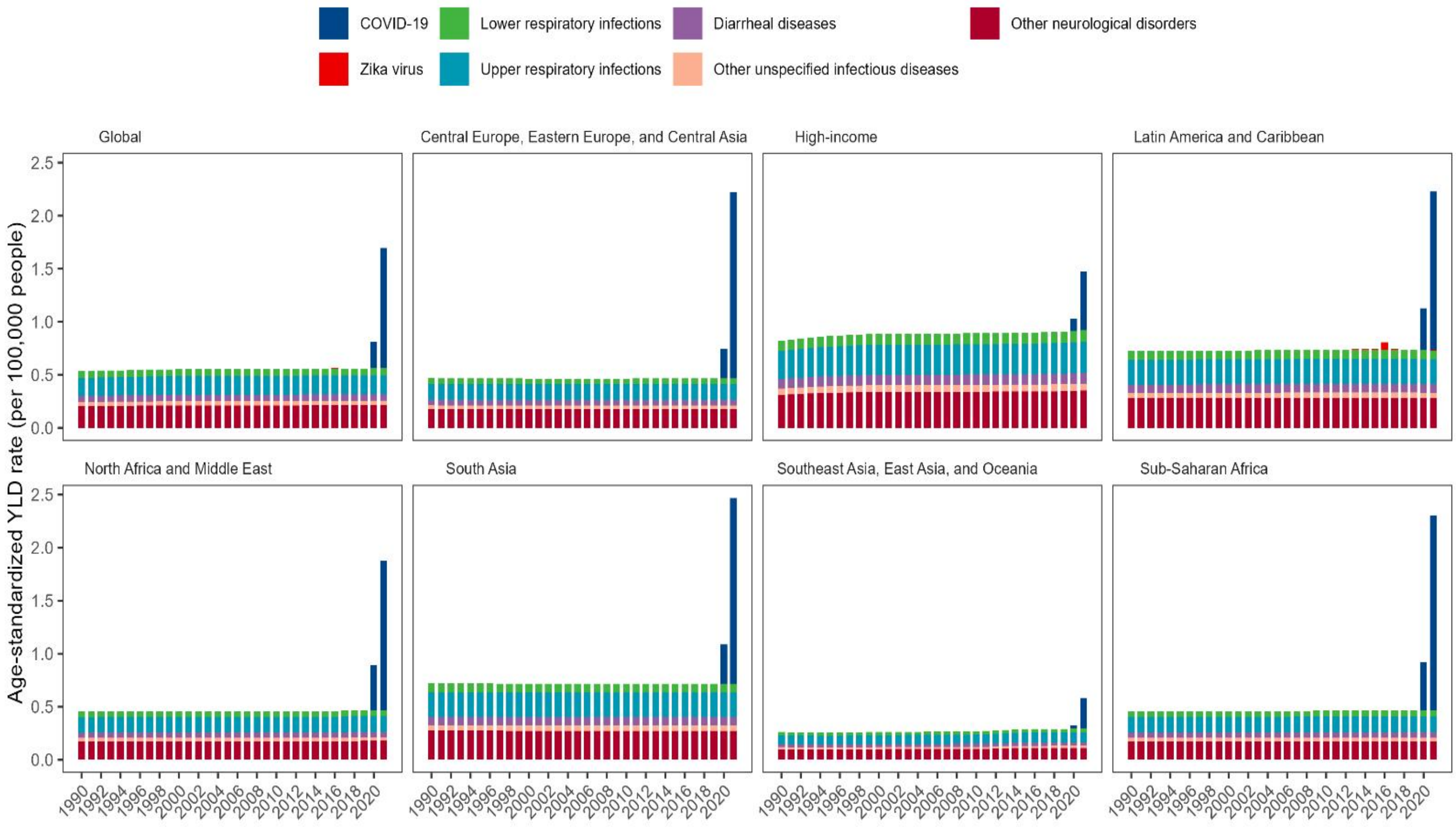

Supplementary Figure 10 Age-standardized years lived with disability rates of Guillain-Barré syndrome attributed to underlying causes in females, by super region and years from 1990-2021.
Notes: Other neurological disorders: idiopathic Guillain-Barré syndrome, the cause is unknown.

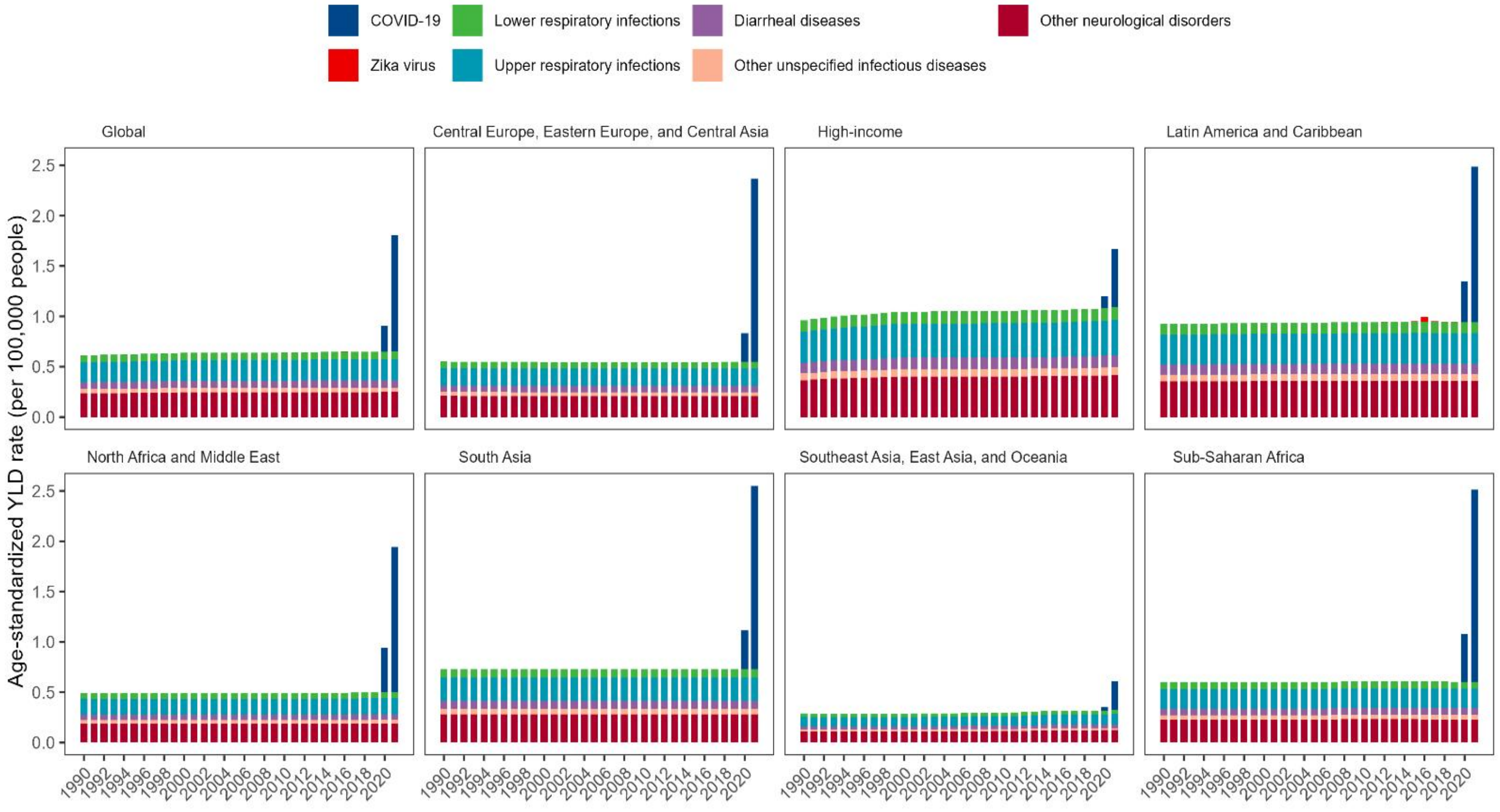

Supplementary Figure 11 Age-standardized years lived with disability rates of Guillain-Barré syndrome attributed to underlying causes in males, by super region and years from 1990-2021.
Notes: Other neurological disorders: idiopathic Guillain-Barré syndrome, the cause is unknown.

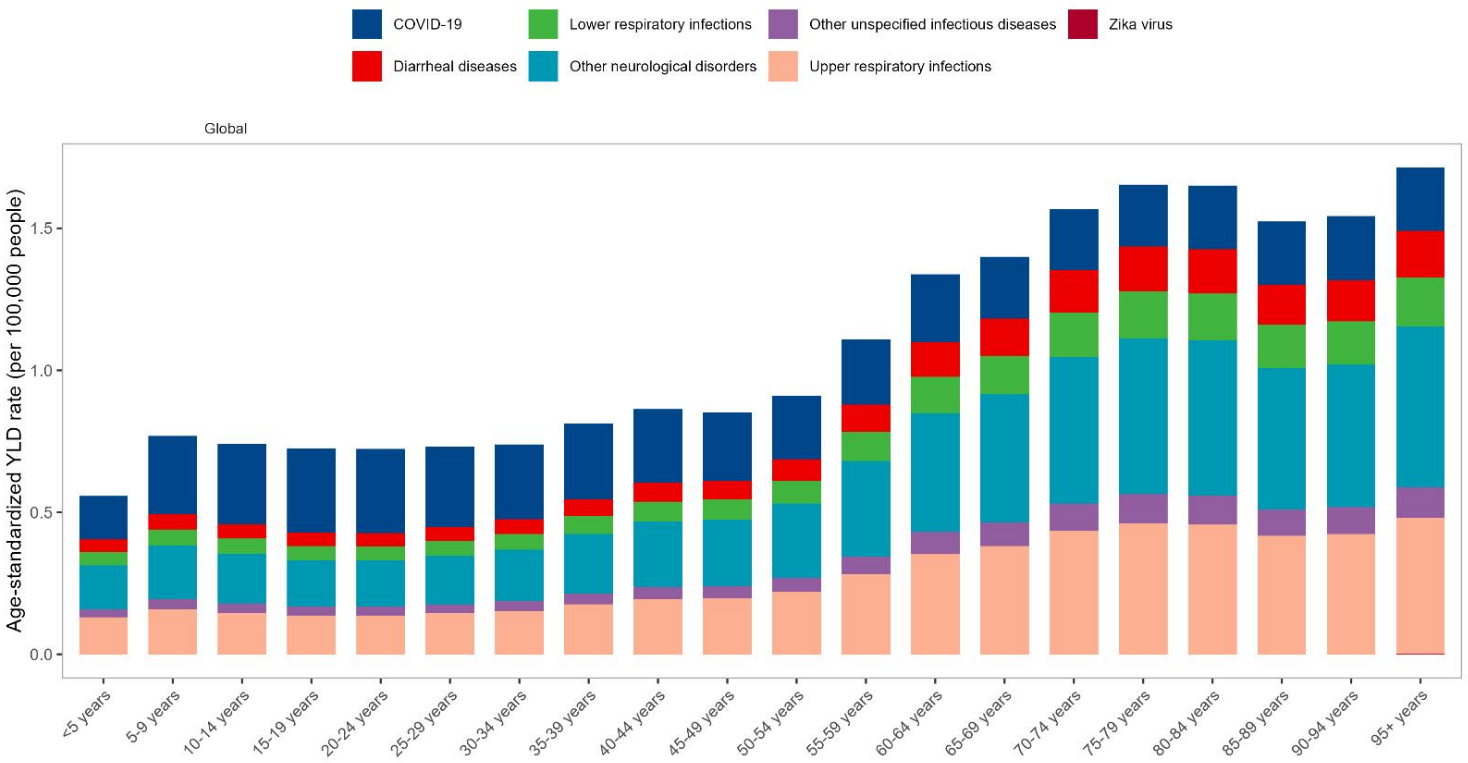

Supplementary Figure 12 Years lived with disability rates of Guillain-Barré syndrome attributed to specific causes by age group in 2020.

Notes: Other neurological disorders: idiopathic Guillain-Barré syndrome, the cause is unknown.

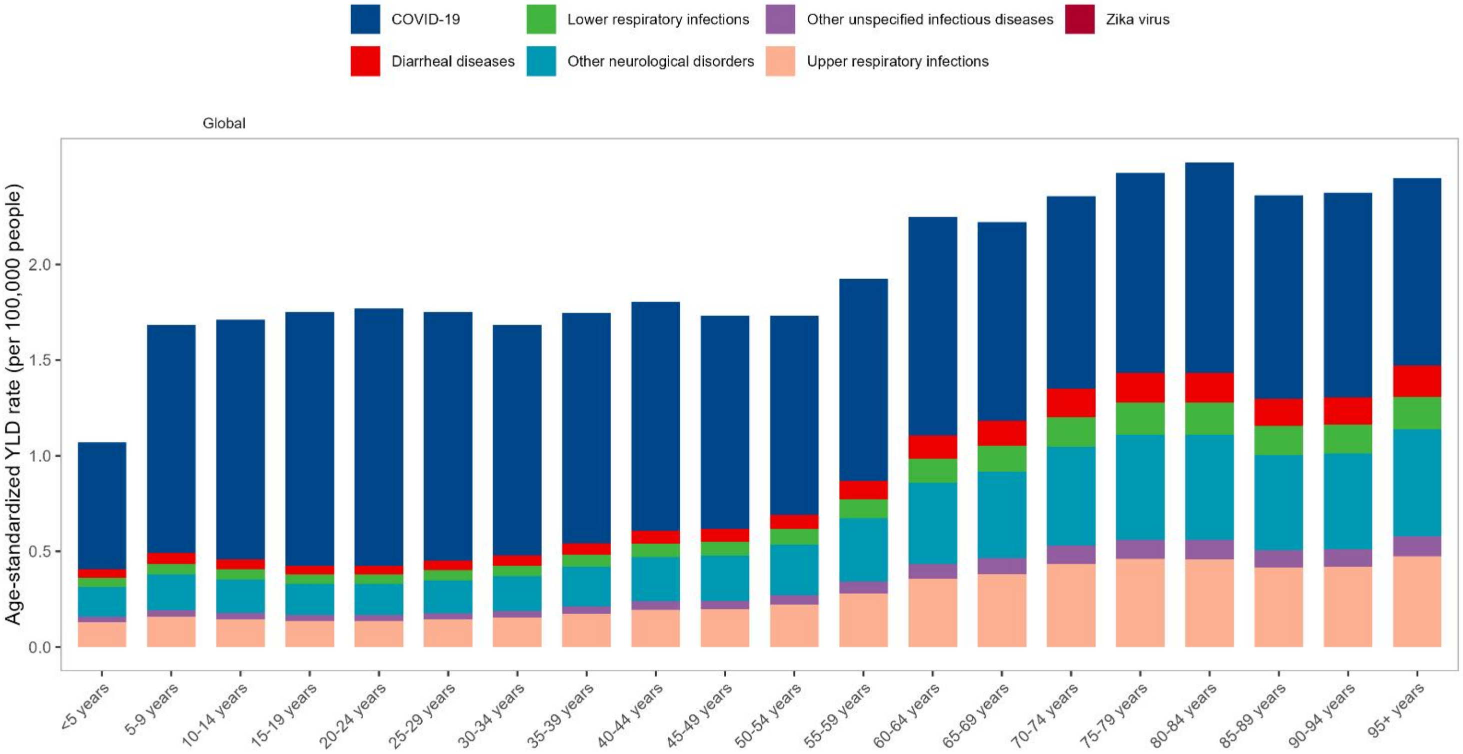

Supplementary Figure 13 Years lived with disability rates of Guillain-Barré syndrome attributed to specific causes by age group in 2021.

Notes: Other neurological disorders: idiopathic Guillain-Barré syndrome, the cause is unknown.

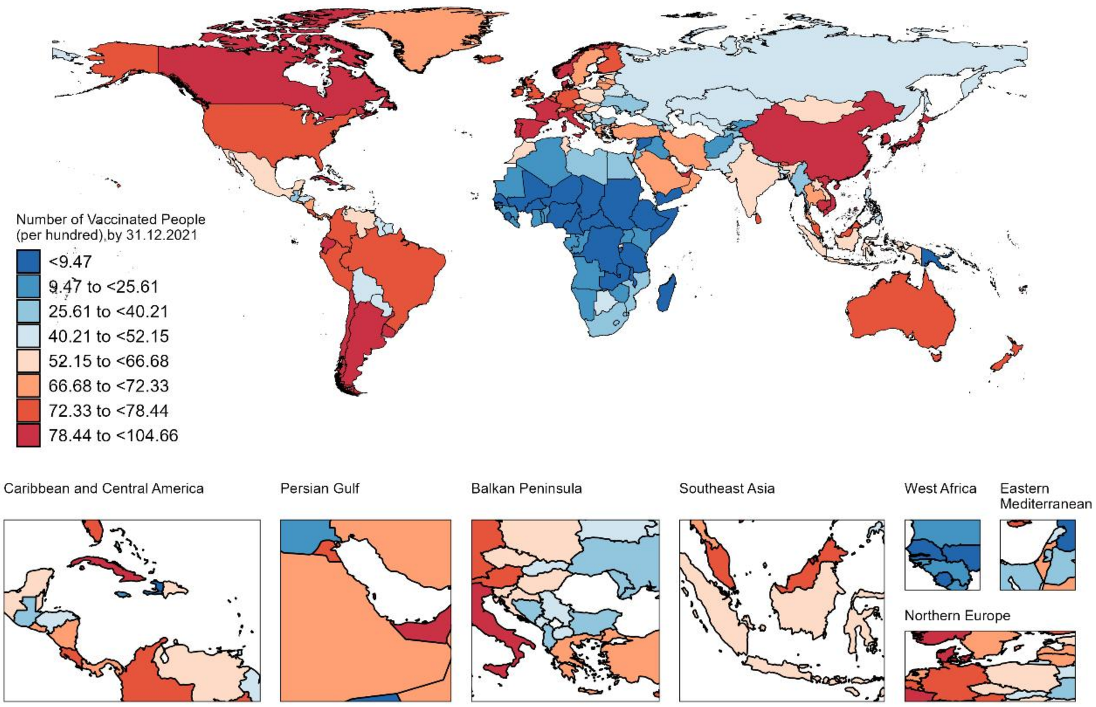

Supplementary Figure 14 Number of people vaccinated per hundred across 181 countries and territories, by 31.12.2021.

The Number of people vaccinated per hundred may be higher than 100% of population. Because changes in population due to migration, birth or death may not be accurately reflected in latest population estimates. If a country has seen increasing migration trends or if a national population statistic is lower than the count recorded in statistical calculations, it can result in a vaccination coverage percentage greater than 100%.

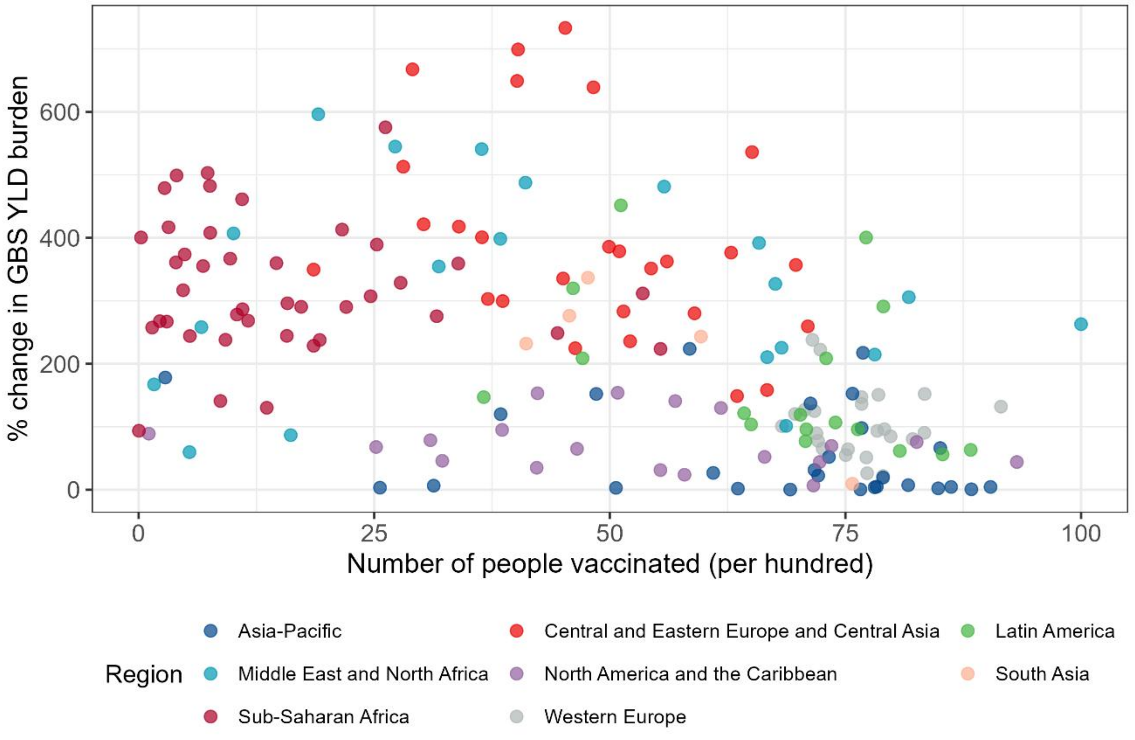

Supplementary Figure 15 Geographic heterogeneity in the relationship between COVID-19 vaccination coverage and GBS YLD change across 181 countries and territories, by 31.12.2021.

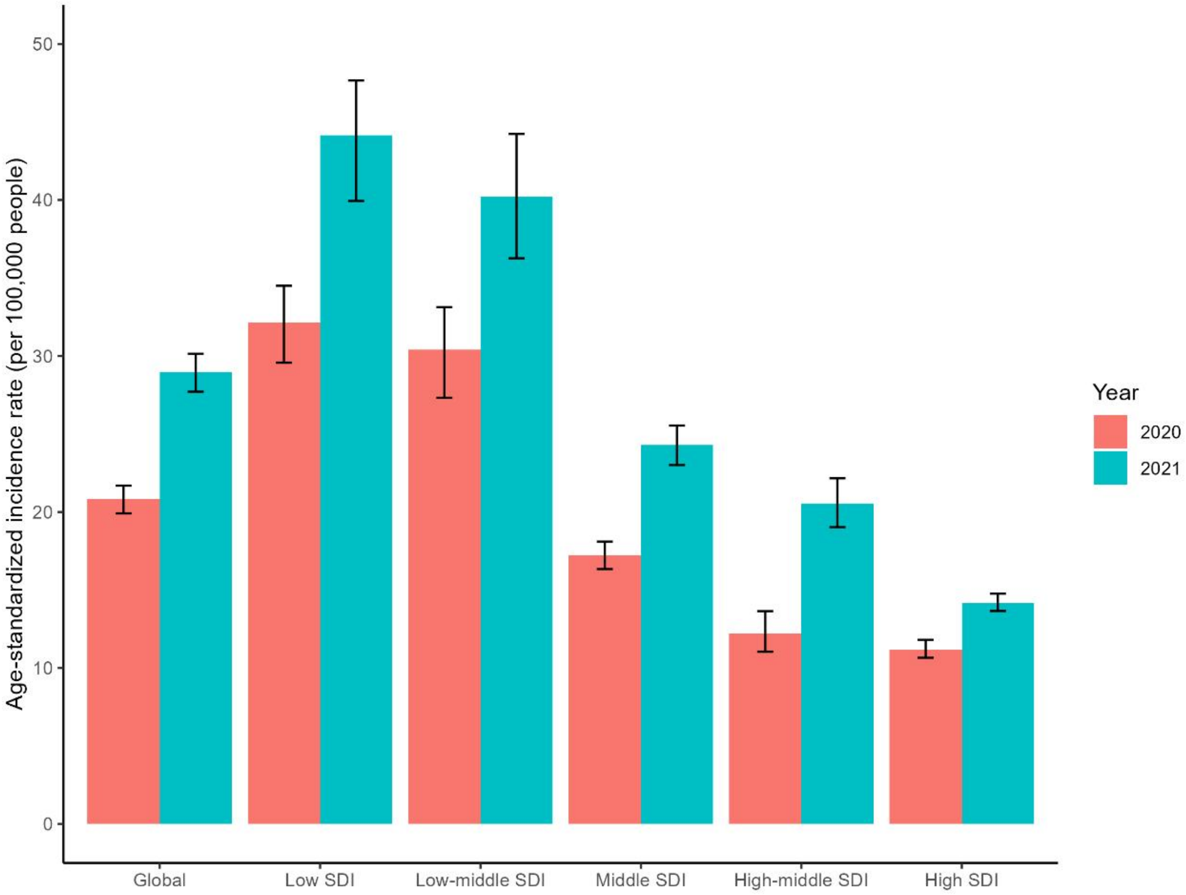

Supplementary Figure 16 Age-standardized COVID-19 incidence by years and socio-demographic index

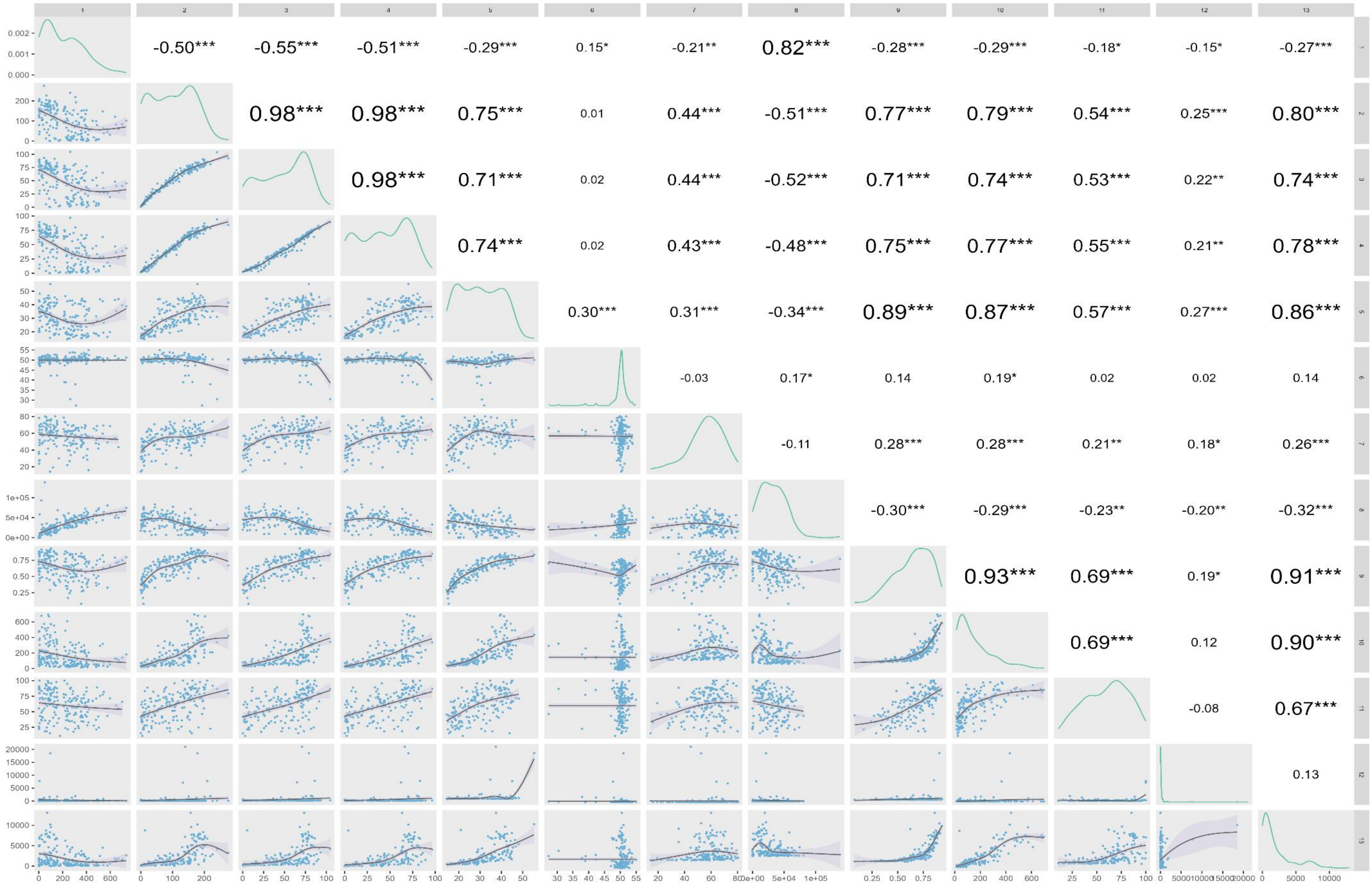

Supplementary Figure 17 Spearman correlation matrix across disease burden of GBS and potential influencing factors.

Notice: 1. Percentage change in YLD rate of GBS, 2019-2021; 2. Total vaccinations (per hundred); 3. People vaccinated (per hundred); 4. People fully vaccinated (per hundred); 5. Median age; 6. Proportion of females; 7. Governance stringency index 2021; 8. COVID-19 incidence (per 100k) in 2021; 9. Social-demographic index levels in 2021; 10. Healthcare human resources density in 2019; 11. Rate of urbanization in 2021; 12. Population density in 2021; 13. Domestic health expenditure in 2021. The curves were fitted using generalized additive model.

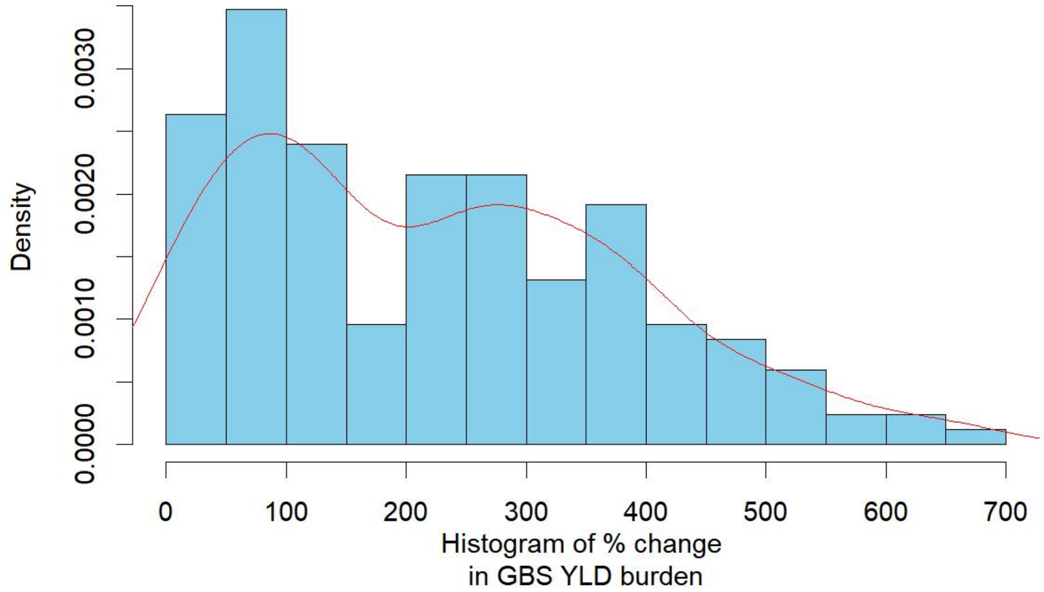

Supplementary Figure 18 Histogram of percentage change in GBS YLD burden without transformation.

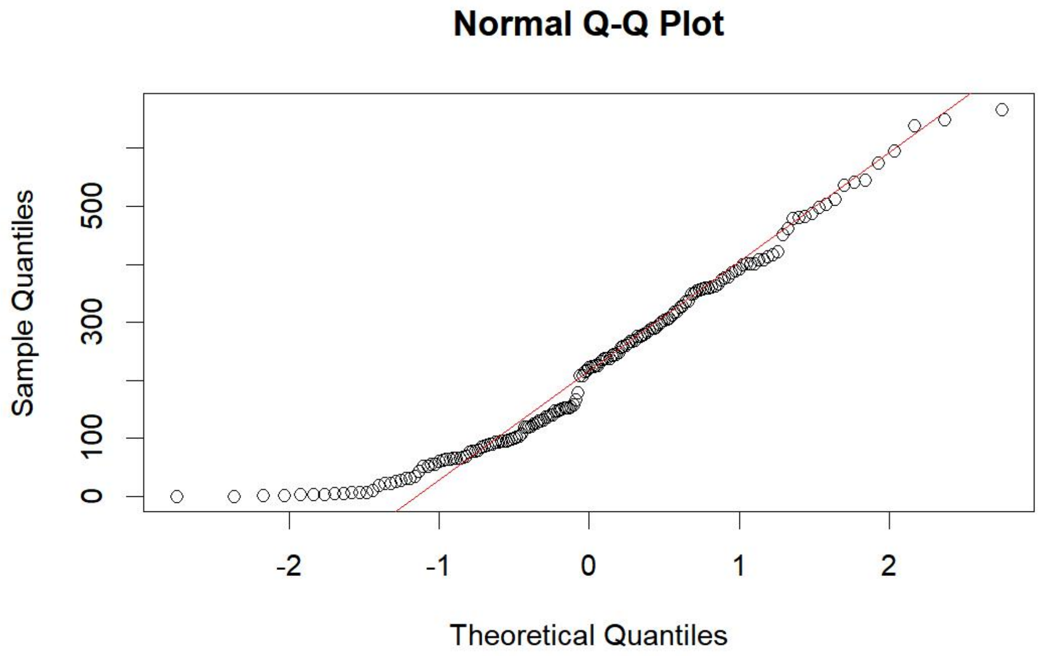

Supplementary Figure 19 Q-Q plot of percentage change in GBS YLD burden without transformation.

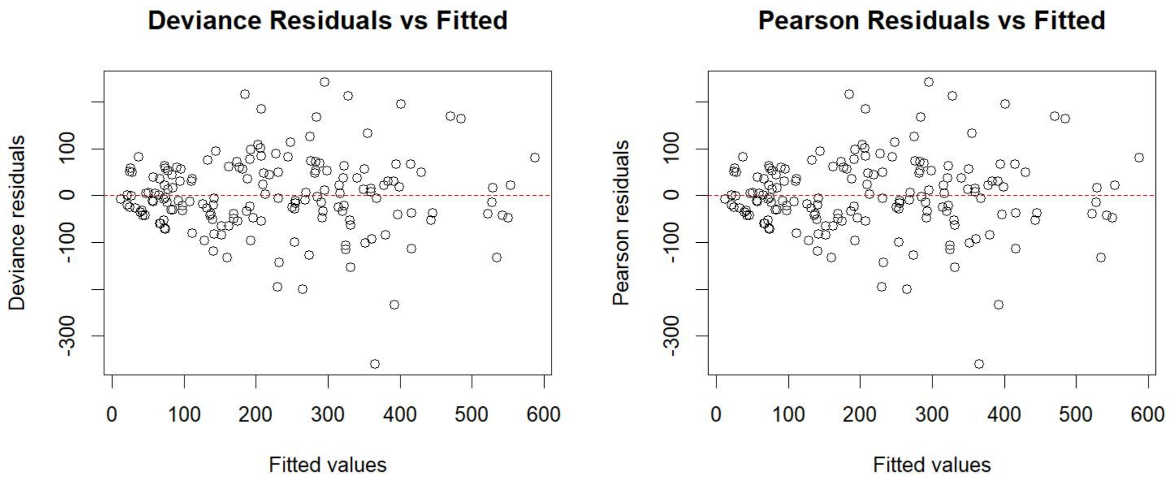

Supplementary Figure 20 Residuals versus fits plot for the GLIM model with Gaussian distribution and log-link function

Note: Shapiro-Wilk test of residual normality (W = 0.98226, p = 0.0304).
